# Seaweed Components as Potential Modulators of the Gut Microbiota

**DOI:** 10.3390/md19070358

**Published:** 2021-06-23

**Authors:** Emer Shannon, Michael Conlon, Maria Hayes

**Affiliations:** 1 Food Biosciences, Teagasc Food Research Centre, Ashtown, D15 KN3K Dublin, Ireland; Maria.Hayes@teagasc.ie; 2 CSIRO Health and Biosecurity, Kintore Avenue, Adelaide, SA 5000, Australia; Michael.Conlon@csiro.au

**Keywords:** seaweed, prebiotics, gut microbiota, polysaccharides, polyphenols, peptides, colonic fermentation, short chain fatty acids, bioaccessibility, simulated gastrointestinal and fermentation digestion models

## Abstract

Macroalgae, or seaweeds, are a rich source of components which may exert beneficial effects on the mammalian gut microbiota through the enhancement of bacterial diversity and abundance. An imbalance of gut bacteria has been linked to the development of disorders such as inflammatory bowel disease, immunodeficiency, hypertension, type-2-diabetes, obesity, and cancer. This review outlines current knowledge from *in vitro* and *in vivo* studies concerning the potential therapeutic application of seaweed-derived polysaccharides, polyphenols and peptides to modulate the gut microbiota through diet. Polysaccharides such as fucoidan, laminarin, alginate, ulvan and porphyran are unique to seaweeds. Several studies have shown their potential to act as prebiotics and to positively modulate the gut microbiota. Prebiotics enhance bacterial populations and often their production of short chain fatty acids, which are the energy source for gastrointestinal epithelial cells, provide protection against pathogens, influence immunomodulation, and induce apoptosis of colon cancer cells. The oral bioaccessibility and bioavailability of seaweed components is also discussed, including the advantages and limitations of static and dynamic *in vitro* gastrointestinal models versus *ex vivo* and *in vivo* methods. Seaweed bioactives show potential for use in prevention and, in some instances, treatment of human disease. However, it is also necessary to confirm these potential, therapeutic effects in large-scale clinical trials. Where possible, we have cited information concerning these trials.

## 1. Introduction

Seaweed-derived components with potential to impact positively on diseases of the body including hypertension [1], cancer [2], type-2-diabetes [3], obesity [4], oxidation [5], inflammation [6] and other disorders have been evaluated in a number of studies to date [7,8,9,10,11,12,13,14,15]. The pathogenesis of these disorders has been linked to the health of the gut microbiota [16]. The microorganisms that inhabit the human gastrointestinal tract—bacteria, archaea, fungi, protozoa, and viruses—are collectively termed the gut microbiota [17]. The gut microbiota is established during infancy [18]. There is a broad variance amongst individuals in microbiota composition because it is shaped by infant transitions such as the gestational period, delivery method, weaning age, breast-feeding duration, or use of formula milk [19]. The microbiota remains relatively stable throughout adulthood but is affected by factors such as enterotype, antibiotic use, diet, lifestyle, genetic traits, and body mass index [20]. Three enterotypes have been described in the human gut microbiome based on variations in levels of the bacterial genera Bacteroides, Prevotella, and Ruminococcus [21]. The gut microbiota is regarded as an endocrine organ that co-develops with the host throughout its life. It exerts an effect on immunity, metabolism, neuroendocrine responses, and synthesises vitamins, amino acids, and enzymes [22,23].

The gut microbiota also aids in the absorption of dietary minerals and produces important short-chain fatty acids (SCFA) such as butyrate, propionate, and acetate. These SCFA are the energy source for gastrointestinal epithelial cells, provide protection against pathogens, influence intestinal mucosal immunity and barrier integrity, and induce apoptosis of colon cancer cells [24,25]. SCFA also regulate liver mitochondrial function, insulin secretion, and induce the production of gut hormones γ-aminobutyric acid and serotonin by interacting with their receptors on enteroendocrine cells [26,27]. An increase in the gut bacterial population enhances the beneficial effects of the microbiota and increases SCFA production [20]. An imbalance or decreased diversity of beneficial versus harmful bacterial species in the gut microbiota is termed dysbiosis and is linked to several diseases [28,29,30,31,32]. Therefore, maintaining the health of the microbiota through diet or supplementary means is thought beneficial to overall health [30]. Seaweed components may exert a beneficial effect on gut health by acting as prebiotics [33,34]. The potential bioactivity of seaweed components has been demonstrated previously in *in vitro* studies [35,36], however the impact of gastrointestinal enzymatic digestion and colonic bacterial fermentation *in vivo* must also be considered, since it may have an effect on the bioavailability of prebiotic and other actives [37,38,39]. As a pharmacological concept, bioavailability is a measure of drug absorption defined as the percentage of the drug that reaches blood circulation, measured by a dose-response curve [40]. However, the evaluation of bioavailability in food-derived extracts differs, since characteristic dose-response curves are not exhibited [41]. In addition, the bioaccessibility of food-derived active compounds must be taken into account, i.e., the accessible portion of the active compound released from the food or extract matrix during digestion [42,43]. Although pharmacokinetic studies are required for the development of prebiotics destined for human and animal use, such studies are not within the scope of this review. The pharmacokinetics of seaweed-derived prebiotics in terms of absorption, distribution, metabolism, and elimination has previously been documented in animal studies after oral administration [44,45,46,47,48] and topical application [49], and recently reviewed by Corino et al. [50] and Shikov et al. [51].

This review outlines current knowledge on the potential beneficial effect of seaweed polysaccharides, polyphenols, and peptides on the gut microbiota and the impact of gastrointestinal digestion and colonic fermentation on their bioaccessibility. The advantages and limitations of static and dynamic gastrointestinal models, and *in vitro*, *ex vivo*, and *in vivo* bioaccessibility and bioavailability assessment methods concerning seaweed bioactives and their prebiotic and potential beneficial health effects are discussed.

## 2. Discussion

Seaweed components that have the potential to exert beneficial effects on the gut by modulating the abundance and diversity of bacterial populations in the gut microbiota include polysaccharides, polyphenols, and peptides. Their structure, function, and studies regarding their potential impact on the gut are considered in this review. Despite the positive results reported from cited studies concerning *in vitro* and animal work, more research is required in human dietary intervention studies, with health-related end points, to determine prebiotic potential.

### 2.1. Polysaccharides 

Polysaccharides, or carbohydrates, are repeating units of monosaccharides linked by glycosidic bonds found in all plants, fungi, and algae. They are considered primary metabolites with structural and energy storage functions [52]. The majority of seaweed polysaccharides are composed of water-soluble and -insoluble fibre [53,54]. The total fibre content of seaweed varies between species and has been reported to range from 35–62% in brown, to 10–57% in red and 29–67% in green (DW) [55,56,57,58,59]. The principal fibres in brown seaweeds are fucoidan, laminarin, and alginate; porphyran, carrageenan, hypnean and floridean starch in red; and ulvan, sulphated-rhamnans, -arabinogalactans and -mannans in green [60,61]. Humans do not produce the endogenous enzymes in the upper gastrointestinal tract required to degrade dietary fibre to monosaccharides. However, fibre is an excellent food substrate, or prebiotic, for human gut bacteria [62,63]. Prebiotics are food components that are indigestible in the small intestine but can be metabolised by microorganisms in the large intestine, modulating their composition and/or activity, thus conferring a beneficial physiological effect on the host [64]. Many species of gut bacteria produce endogenous carbohydrate-degrading enzymes, such as β-glucanase and β-glucosidase, capable of hydrolysing the glycosidic linkages of polysaccharides [65,66,67,68]. Several polysaccharides within seaweed that are indigestible in the upper gastrointestinal tract are thought to exert bioactive effects including glycaemic control [69] and the promotion of gut microbial- and immune-modulation by acting as prebiotics in *in vitro* and *in vivo* studies [70,71]. The bioactivity of polysaccharide fractions is influenced by a number of factors such as chemical structure, molecular weight (MW), solubility, extraction method, seaweed genus and seasonal variation [72,73]. The principal polysaccharides of brown, red, and green seaweeds are detailed below.

#### 2.1.1. Fucoidans

Three polysaccharides—fucoidans, laminarin and alginate—occur within brown seaweeds, each of which have differing structures and functions [74]. Fucoidans comprise 5–20% (DW) of the entire seaweed thallus [75,76]. They are water-soluble sulphated-polysaccharides composed of repeating fucose and sulphate groups, and may also contain galactose, mannose, xylose, rhamnose, arabinose, glucose, acetyl groups, or glucuronic acid [77]. The molecular weight of fucoidans varies from 7 to 2300 kDa [11]. Fucoidans provide structure for the outer cell wall and a hydrophilic coating to prevent desiccation of the seaweed during low tide. They also play a role in adapting to osmotic stress caused by changes in salinity as their sulphate groups can bind to cations such as sodium, potassium, magnesium, and calcium [78,79]. Fucoidans have previously been shown in *in vitro* studies to have potential for use as anticancer [80], antiviral [81], antioxidant [77], and anti-inflammatory [82] agents; and *in vivo* as anticoagulants (human trial) [83], anticancer (human trial) [84], antitumour (mouse model) [85], antihyperglycaemic, and antihyperlipidaemic agents (mouse model) [86]. However, the oral bioavailability of fucoidan can be low due its highly polar nature and limited ability to pass through intestinal epithelial cells [68]. In recent years, the prebiotic status of fucoidan has been recognised *in vitro* [82,87] and in human [88] and animal [89,90,91,92] gastrointestinal studies. 

#### 2.1.2. Laminarin 

The energy storage polysaccharide of brown seaweeds is laminarin, composed of β(1–3)-linked glucose units with β(1–6)-branches [93]. It occurs within the chloroplasts in micro-compartments called pyrenoids [94]. Laminarin is water-soluble, though increased branching of the molecule requires colder temperatures for solubility. It comprises 3–35% of brown seaweed dry mass and is most prevalent in Laminaria species [95]. It is a small polysaccharide with a molecular weight of approximately 5 kDa [96]. Laminarin has shown efficacy in *in vitro* studies carried out previously and has potential for use as an anticancer [97], antimetastatic [98], antioxidant [99] and immunostimulatory [100] agent [97,99,100]; and *in vivo* as an immunomodulatory agent [101] and prebiotic to modulate dysbiosis (animal models) [102,103,104,105].

#### 2.1.3. Alginate 

Alginate comprises up to 45% of brown seaweed dry mass [106], occurring in the cell walls as salts of alginic acid bound to sodium, calcium or magnesium ions [107]. It is a water-soluble linear polysaccharide composed of (1–4)-linked β-D-mannuronate and α-L-guluronate residues [108]. Molecular weight ranges from 20 to 350 kDa [109,110]. It is the most abundant polysaccharide in brown seaweed and imparts flexibility to the thallus to withstand the force of the ocean. Alginate is a phycocolloid that can bind up to 20 times its own mass with water, making it very useful for food and industrial applications [111]. The prebiotic effect of alginate on gut microbiota was demonstrated previously *in vitro* by Bai et al. [112] and Li et al. [113]; and in a human study by Mizuno et al. [114]. Bai et al. fermented seaweed-derived alginates *in vitro* and observed that the alginates were degraded by human-derived gut bacteria, producing a significant (*p* < 0.05) increase in SCFA compared to a starch control, and suggested that further investigations of the prebiotic effects of alginate are warranted. Li et al. also fermented seaweed-derived alginates with human faecal bacteria *in vitro* and found a significant (*p* < 0.05) increase in total SCFA in the alginate sample (78.6 ± 5.9 mM) compared to the control (62.5 ± 5.1 mM). The bacterial Richness index in the alginate ferment (15.83 ± 2.3) was also significantly greater (*p* < 0.05) than that of the control (12.67 ± 2.88). The authors propounded that alginate may be capable of sustaining the growth of human gut bacteria, and recommended further study to evaluate the potential impact that alginate food additives may exert on host health. The *in vivo* study by Mizuno et al. was an interventional study of 11 elderly patients who required enteral feeding. After 4 weeks of receiving the alginate formula (equivalent to 14.52 g fibre/day) there was a significant increase (*p* = 0.039) in Clostridium cluster XI bacteria compared with the baseline. However, there was no increase in Bifidobacterium, Lactobacillales, or Bacteroides. The patients’ stool form improved (*p* = 0.044) (Bristol Stool Scale), as did mean blood concentrations of total SCFA (*p* = 0.042), acetic acid (*p* = 0.042), propionic acid (*p* = 0.027), serum albumin (*p* = 0.039), total cholesterol (*p* = 0.002), and cholinesterase (*p* = 0.034). The alginate did not induce any significant changes in stool frequency, body weight, or arm circumference. The authors suggested that the alginate-containing liquid formula may potentially exert a beneficial prebiotic effect on intestinal function through increased production of SCFA. However, the limitations of the study were noted due to the small sample size and single-center study design. In order to validate the findings, the authors recommend a larger, multicenter study.

Alginate may also be useful in the prevention of metabolic syndrome syndrome [115]. It can increase the viscosity of gastric contents, reducing postprandial glucose absorption and insulin response [116], and may thereby impact on hyperlipidaemia and hypertension [1,117].

#### 2.1.4. Carrageenans

Within red seaweeds, carrageenans and porphyran are the prevalent polysaccharides. The family of linear, sulphated polysaccharides, carrageenans, occur as a structural component of the extracellular matrix [118]. Of the 15 different carrageenan forms, iota (ι), kappa (κ) and lambda (λ) are the most widely used as phycocolloids in the food industry [119] and as a vegan alternative to beef gelatin in pharmaceutical capsules [120]. κ and ι-carrageenan are composed of alternating d-galactose and 3,6-anhydro-galactose units with varying numbers of sulphate groups, while λ-carrageenan lacks 3,6-anhydro-galactose and has alternating α-1,3 and β-1,4 inter-galactose bonds [121]. Average molecular weight ranges from 453 to 652 kDa [122]. All forms of carrageenan are soluble in water above their gel-melting temperatures (40–70° C). In cold water, only λ-carrageenan and the sodium salts of κ and ι-carrageenan are soluble [93]. ι-carrageenan was shown to reverse the symptoms of metabolic syndrome in a rat model by significantly decreasing systolic blood pressure, body mass (BM), abdominal and liver fat, and total cholesterol, while also beneficially modulating the gut microbiota [123]. As potential antitumour agents, κ/ι hybrid carrageenans have shown activity *in vitro* against colorectal cancer stem cell-enriched tumourspheres [2]. However, simulated gastrointestinal studies have found that κ-carrageenan can be both beneficial and harmful by increasing or decreasing markers of inflammation and the growth of beneficial gut bacteria and SCFA. This is dependent on the degree of polymerisation of the carrageenan [124].

#### 2.1.5. Porphyran

Porphyran is a sulphated polysaccharide that occurs in red seaweed, within the genus Porphyra, and comprises approximately 11–21% of the seaweed dry mass [125]. It is composed of repeating units of galactose and 3,6-anhydrogalactose, with alternating units of galactose-6-sulphate and 6-*O*-methyl-galactose [126]. Average molecular weight ranges from 14 to 201 kDa [127,128]. Porphyran is soluble in hot water and has similar structural functions to carrageenan, though its higher viscosity limits its pharmaceutical applications [128,129]. Porphyran has shown potential antioxidant and anti-inflammatory effects in cell studies using RAW264.7 cell line [125] and was found to promote cell migration and proliferation in intestinal epithelial cells [127]. It also has antitumor activity against HeLa cells [130], HT-29 colon cancer cells and AGS gastric cancer cells [131]. As a prebiotic, porphyran was previously found to increase beneficial gut bacteria and SCFA production *in vitro* in simulated digestion studies [126,132,133] and in animal studies as whole red seaweed [134,135,136].

#### 2.1.6. Ulvans 

Green seaweeds are dominated by the ulvans, which account for 38–54% of the thallus dry mass [137]. Ulvans are water-soluble, gelling polysaccharides composed of repeating units of sulphated l-rhamnose, d-xylose, d-glucuronic acid and its epimer L-iduronic acid [138]. Molecular weights range widely from 1 to 2000 kDa depending upon the degree of sulphation [139]. Ulvans have demonstrated potential anticoagulant [140], antibacterial [141], antiviral [142], and immunoregulatory (porcine intestinal epithelial cells) [143] activities *in vitro*. They have also shown potential for the use as prebiotics in animal studies [144] and *in vitro* [132,145,146].

### 2.2. Gastrointestinal Digestion Studies with Seaweed Polysaccharides

A number of recent studies have used simulated *in vitro* gastrointestinal digestion or *in vivo* clinical trials to investigate the effect of polysaccharides on beneficial bacterial populations and their metabolites. Table 1 summarises the polysaccharide fraction used in each study and its impact on gut bacteria. Further characterisation and *in vivo* animal and human dietary intervention studies are required to confirm any potential therapeutic benefits.

### 2.3. Polyphenols

Polyphenols are secondary metabolites that occur ubiquitously in terrestrial plants and algae. They are composed of repeating units of phenol—an aromatic phenyl group (a benzene ring, minus one hydrogen atom) bound to one or more hydroxyl groups [152]. Polyphenols are involved in numerous functions. They protect the seaweed thallus against biotic and abiotic stresses such as predation from herbivores, microbial infection, oxidation, and UV damage [153]. The total polyphenolic content of brown seaweed (dry mass) can be as high as 20%, while green and red seaweeds contain 1–5% [154,155,156]. The molecular weight of seaweed polyphenols ranges from approximately 26 Da to 650 kDa [157,158]. Seaweed polyphenols have been found to increase high-density lipoprotein cholesterol [159], post-prandial cognitive function [160], and exert anti-hypertensive [161] anti-hyperglycaemic [162] and peak blood glucose reducing effects (females only) [163] in human studies. However, only 5–10% of polyphenols are absorbed in the upper gastrointestinal tract due to their structural complexity. Large polyphenol compounds that reach the large intestine can be converted by microbial activity into beneficial bioactive metabolites [164,165], while also inhibiting pathogenic species [166]. Gut microbial enzymes catabolise polyphenols via hydrolysis, dehydroxylation, decarboxylation, reduction, demethylation, and isomerisation [167]. Studies with germfree animals have shown that bioactive phenolic metabolites—normally found after oral administration of polyphenols—are absent in their gut [168]. This shows the importance of the gut microbiota in polyphenol metabolism.

In terrestrial plants, the predominant polyphenols are flavonoids, stilbenes, lignans, and phenolic acids [169]. Seaweeds also produce flavonoids, coumarins, phenolic terpenoids, phenolic acids, luteolin, regiolone, and neoeriocitrin as well other polyphenols that are unique to algae [170,171,172]. These include bromophenols and phlorotannins [173].

#### 2.3.1. Bromophenols

Bromophenols are molecules composed of one to five phenol groups, bound to one or more bromine [174]. Bromophenols are produced by seaweed as part of their chemical defence system to protect them from herbivores [175], oxidation, bacteria, and fungi [176,177]. Tri-bromophenols are the most common isomers found in seaweed, followed by di- and mono- bromophenols [178]. Bromophenols occur most abundantly in red and green seaweeds, and to a lesser extent in brown genera. A study of 49 red, green, and brown seaweeds by Whitfield et al. [179] reported bromophenol contents ranging from 8 to 2590 ng/g in red, 0.9 to 2393 ng/g in green, and 2 to 454 ng/g in brown. Seaweed-derived bromophenols have antioxidant [180], anti-inflammatory [181], antibacterial [182], anti-cancer [183], antithrombotic [184], and antidiabetic [185] activity.

#### 2.3.2. Phlorotannins

Phlorotannins, found only in brown seaweeds, are composed of repeating units of phloroglucinol—a phenyl ring bound to three hydroxyl groups. Due to their ability to precipitate proteins, they are considered tannins [186]. Phlorotannins have structural functions within the seaweed cell wall [187], and protect against oxidation [188] and predation by herbivores [189]. Phlorotannins are sub-classified into four main groups depending upon the type of chemical bonds that link their phloroglucinol units [190]. Fuhalols and phlorethols have ether bonds; fucols have phenyl bonds; fucophlorethols have phenyl and ether bonds; while eckols have dibenzodioxin bonds [191]. The molecular weight of phlorotannins ranges broadly depending upon the number of phloroglucinol units they contain [192]. Molecular weights have been reported from 1.2 to 6 kDa [193], 30 to 100 kDa [194], and as high as 300 kDa [195]. Phlorotannin content differs broadly amongst species, and is influenced by seasonal variations and geographic location [196,197]. Content is generally expressed as gallic acid or phloroglucinol equivalents, or as a percentage of seaweed dry mass. A study of eight brown seaweeds over 14 months from the same location in France by Connan et al. [196] reported significant inter- and intra-species seasonal differences in phlorotannin content, with the highest values occurring in summer. Values ranged from 0.13% phlorotannin content (DW of total seaweed) in *L*. *digitata*, to 5.80% in *A*. *nodosum* and *F*. *vesiculosus*. Phlorotannins have been studied for their potential health effects. Reported bioactivities include antioxidant [198], antidiabetic [199], anticancer [200], antihypertensive [201], anti-inflammatory [202], antiviral [203], neuroprotective [204], antimicrobial [205], and prebiotic activities [10,206,207].

### 2.4. In Vitro and In Vivo Gastrointestinal Digestion Studies with Seaweed Polyphenols

The effect of polyphenols, particularly phlorotannins, on the gut, metabolic syndrome, and DNA damage has been reported in some *in vitro* and *in vivo* studies which are discussed below. 

#### 2.4.1. Prebiotic Function and Attenuation of Metabolic Syndrome by Phlorotannins

Charoensiddhi et al. [10] evaluated the prebiotic potential of phlorotannin enriched (PE) ethanolic extracts *in vitro* from *E. radiata* harvested in Australia. After 24 h fermentation, the phlorotannin extracts induced significant increases (all *p <* 0.05) in some populations of beneficial bacteria, which were selected for the study due to their relevance to gut health [10]. These were: Bacteroidetes (6.52 ± 0.04 log_10_ cells/mL) compared to the cellulose control (6.40 ± 0.05 log_10_ cells/mL); *F. prausnitzii* (6.57 ± 0.05 log_10_ cells/mL) compared to inulin and cellulose controls (6.17 ± 0.04 and 6.07 ± 0.06 log_10_ cells/mL, respectively); *C. coccoides* (7.97 ± 0.05 log_10_ cells/mL) compared to inulin and cellulose controls (7.57 ± 0.06 and 7.40 ± 0.05 log_10_ cells/mL, respectively); and *E. coli* (8.09 ± 0.02 log_10_ cells/mL) compared to inulin and cellulose controls (6.81 ± 0.03 and 6.94 ± 0.03 log_10_ cells/mL, respectively). However, the production of SCFA was not enhanced by fermentation with the phlorotannin extract. 

Lin et al. [170] reported the effect of a polyphenolic extract from the green seaweed, *Enteromorpha prolifera*, harvested in China, on the gut microbiome and glucose metabolism of diabetic mice. Polyphenols were extracted from *E. prolifera* using ultrasound-assisted ethanol and ultrafiltration to a MW of 3 kDa. The extract was characterised by UPLC-MS and found to contain four polyphenols—luteolin-6-c-glucoside, regiolone, neoeriocitrin, and estr-5(10)-ene-3,17-diol. Diabetes was induced in ICR mice (20/group) using STZ. Ten of the diabetic mice received a high-sucrose/high-fat diet with no polyphenol supplement (model group); while 10 received a high-sucrose/high-fat diet with *E. prolifera* polyphenol extract (300 mg/kg BM/d) (diabetic group). A control group of non-diabetic mice received standard chow (normal group). 

After 28 days, there was an increase (*p <* 0.05) in the abundance of beneficial Alistipes intestinal bacteria in the polyphenol-fed diabetic group compared to the model group. After 14 days, there was a significant reduction (*p <* 0.05) in the mean BM of the *E. prolifera*-fed diabetic group compared to the model group. After 28 days, fasting blood glucose levels of the diabetic group were lower (*p <* 0.05), and glucose tolerance was increased (*p <* 0.05) compared to the model group. 

Histopathological analysis of the liver revealed that the polyphenol-fed diabetic group had less cell damage and inflammation of the hepatic cord than the model group. The mRNA expression of two proteins associated with glucose metabolism was also measured in liver tissue—phosphatidylinositol 3-kinase (PI3K) and c-Jun N-terminal kinase (JNK). The PI3K pathway regulates insulin signal transduction and glucose homeostasis [208], while over-activity of the JNK pathway is linked to insulin resistance and type-2-diabetes [209]. After 28 days, mRNA expression of PI3K was increased in the diabetic group (*p <* 0.01) compared to the model group, and was even significantly higher than the normal group (*p <* 0.05). JNK1 expression in the diabetic group was successfully downregulated by polyphenol supplementation and was lower (*p <* 0.05) than the model group.

Yuan et al. [210] investigated the ability of polyphenol extracts from the brown seaweed, *Lessonia trabeculata*, harvested in China, to alter the gut microbiota of rats in response to type-2-diabetes. Microwave-assisted methanol extraction was followed by solvent fractionation and macroporous resin adsorption separation. The polyphenol-rich fractions produced were composed primarily of phlorotannins, followed by phenolic acids and gallocatechin derivatives. Diabetes was induced in C57BL/6J rats using streptozotocin (STZ). STZ damages the insulin-producing β cells of the pancreas, resulting in hypoinsulinaemia and hyperglycaemia. Diabetic rats (8/group) (PE) were fed 200 mg/day polyphenol extract/kg BM along with their regular food for 4 weeks. A diabetes control (DC) group and a normal control (NC) group (of non-diabetic rats) received no polyphenol supplement with their food. 

Hyperglycaemia, insulin resistance, and hyperlipidaemia were significantly (*p <* 0.01) reduced in the diabetic rats after 4 weeks administration of the seaweed polyphenol extract. Mean fasting blood glucose was lower (*p* < 0.05) in the PE group (10.55 ± 0.94 mmol/L) compared to the DC control group (13.99 ± 0.87 mmol/L) as was serum insulin (14.69 ± 0.11 vs. 17.70 ± 0.22 mU/L (*p* < 0.01)). The homeostatic model assessment of insulin resistance (HOMA-IR) value was lower in the PE group (*p* < 0.01) (6.89 ± 0.42 vs. 11.01 ± 0.98) compared to the DC group. The reductions in lipid profiles in the PE group compared to the DC group were: total cholesterol (4.92 ± 0.14 vs. 5.64 ± 0.16 mmol/L (*p* < 0.01)), triglycerides (0.99 ± 0.04 vs. 1.43 ± 0.10 mmol/L (*p* < 0.01)), low-density lipoprotein cholesterol (0.68 ± 0.03 vs. 1.06 ± 0.06 (*p* < 0.0)), glycated serum protein (2.15 ± 0.16 vs. 2.74 ± 0.15 (*p* < 0.01)) and non-esterified fatty acids (1.86 ± 0.05 vs. 2.02 ± 0.11 mmol/L (*p* < 0.05)). The dyslipidaemia observed in the DC group who did not receive polyphenol supplementation was most likely due to the deficiency of circulating insulin, which increases lipase activity and fatty acid mobilisation from adipose tissue [211]. 16S rRNA gene sequencing of faecal samples from the diabetic rats revealed that there was a significant (*p <* 0.01) increase in gut bacterial diversity within the polyphenol-fed PE group compared to the DC and NC groups. The PE group had a significantly greater abundance of Bacteroidetes, less Proteobacteria, and an improved (lower) ratio of Firmicutes to Bacteroidetes compared to DC (*p* < 0.01). An overabundance of Proteobacteria has been reported as a pro-inflammatory phylum and linked with the imbalance of glucose homeostasis in type-2-diabetes [170]. At the genus level, the PE group had approximately 10 times more Odoribacter (*p <* 0.008) and Muribaculum (*p <* 0.005), and twice the population of Alistipes (*p <* 0.006), Lachnospiraceae (*p <* 0.015) and Parabacteroides (*p <* 0.022) compared to the DC group. Lachnospiraceae and Alistipes are butyric acid producing bacteria that contribute to the maintenance of colonic epithelial tissue [212]. The Odoribacter genus, part of the Bacteroidetes phylum, is an acetic, propionic and butyric acid producer. Its abundance ameliorates inflammation by increasing SCFA availability [213]. An increase in Muribaculum and Parabacteroides numbers has been reported to combat dyslipidaemia, weight gain, inflammation, and insulin resistance resistance [214,215]. 

Quantification of gut SCFA showed a 61.1% increase in total SCFA production (from 491.31 ± 10.39 to 1276.34 ± 16.86 μg/g (*p* < 0.01)) by the rats after 4 weeks of polyphenol supplementation. The PE group also produced 68.6% more acetic acid (1202.49 ± 11.55 compared to 377.77 ± 3.46 μg/g (*p* < 0.01)) and 74.4% more butyric acid (39.77 ± 1.85 compared to 10.18 ± 0.58 μg/g (*p* < 0.01)) than the DC group. The authors of the study concluded that seaweed polyphenols may have regulated dysbiosis of the gut microbiota in diabetic rats.

#### 2.4.2. Impact of Digestion on Phlorotannin Bioactivity, Attenuation of DNA Damage, and Cancer Cell Proliferation In Vitro

Corona et al. [216] studied the effect of *in vitro* gastrointestinal digestion and colonic fermentation on the polyphenolic content and bioactivity of high molecular weight (HMW > 10 KDa) and low molecular weight (LMW 1–10 KDa) ethanol-extracted phlorotannins from *A*. *nodosum* harvested in Scotland. To assess changes in phlorotannin bioactivity post-gastric digestion and -fermentation, the ability of the extracts to prevent H_2_O_2_ induced DNA damage in HT-29 colon cancer cells and inhibit cell proliferation was also measured. The HMW extract had the greatest total polyphenol and total phlorotannin contents before and after digestion. The HMW extract also had the highest Trolox equivalent antioxidant capacity. The molecular weight of total phlorotannins before and after gastric digestion and colonic fermentation was evaluated by normal phase HPLC. Gastric digestion reduced the level of very high molecular weight components present in the HMW fraction by only 5.4%, while colonic fermentation caused an 89.9% reduction. In the LMW extracts, gastric digestion reduced the level of very high molecular weight components by 52.8% and colonic fermentation by 62.0%. In both cases, colonic fermentation had a far greater impact on the breakdown of phlorotannins compared to enzymatic gastric digestion, suggesting that phlorotannins have the potential to be metabolised by human gut bacteria. 

A sulforhodamine B assay was used to measure changes in HT-29 colon cancer cell biomass. The addition of post-gastric digested HMW and LMW at a concentration of 500 μg/mL significantly inhibited (*p <* 0.01) HT-29 cell proliferation (number of cells by division), with HMW being the most effective. Post-gastric digested LMW did not inhibit cell growth (mass accumulation) at any concentration, but HMW did (*p <* 0.05) at concentrations of 250 and 500 μg/mL. High molecular weight phlorotannins may therefore have a potential protective effect on colonocytes against cancer. H_2_O_2_ induced DNA damage in HT-29 cells was evaluated by single cell gel electrophoresis (Comet) assay. Three of the four phlorotannin extracts (at 100 μg/mL) were successful in reducing DNA damage. Post-gastric digested HMW significantly (*p <* 0.01) reduced DNA damage compared to the control, while post-gastric digested LMW had no effect. However, both the HMW and LMW post-colonic fermented extracts significantly (*p <* 0.001) reduced DNA damage, suggesting that colonic bacteria may potentially metabolise phlorotannins into molecules with different bioactivity than their parent structures. 

Although *in vitro* studies and animal trials do not replicate the human gut environment identically, these results show that the abundance of bacteria which normally colonise the mammalian gut may potentially be enhanced by the inclusion of dietary polyphenols. The findings are an indication of prebiotic potential, which may be used to inform the design of future human clinical studies. Table 2 summarises the polyphenol used in each study and its potential impact on the gut microbiota *in vitro* and *in vivo*, the modulation of hyperglycaemia in animal models, and attenuation of DNA damage *in vitro*.

### 2.5. Seaweed-Derived Peptides

Seaweed-derived peptides have reported bioactivity as inhibitors of renin [217], angiotensin converting enzyme-I (ACE-I) [9] dipeptidyl peptidase (DPP-IV) [218], platelet activating factor acetylhydrolase (PAF-AH) [219] and α-amylase [220]. They also have reported immunostimulatory [221], antitumor [222], anti-coagulant [223], antioxidative [5], and anti-hyperglycaemic [224] activity. There is recent evidence that some peptides found *in vitro* correlate with animal studies [225]. A study conducted by Fitzgerald et al. [217] previously identified the potential heart health beneficial effects of peptides included in a bread product with peptides derived from the red seaweed *Palmaria palmata* using both *in vitro* and animal models [226]. Peptides were isolated from the seaweed and characterised to completion. However, another study carried out by Allsopp et al. [227] found that the same seaweed had a pro-inflammatory effect when consumed as a whole seaweed in a bread product. This highlights the importance of extraction and characterisation of seaweed bioactives for potential use as therapeutic agents. Allsopp et al. suggested that the iodine content of the seaweed may have been responsible for the observed pro-inflammatory effect in a human dietary intervention study. 

Table 3 details the amino acid sequences of recently elucidated seaweed peptides and their bioactivities *in vitro*, *in silico*, or *in vivo*.

Proteins and peptides can be used as a food substrate by some families of colonic bacteria including Enterobacteriacea, Burkholderiacea, and Desulfovibrionacea [232] and the genera Peptostreptococcus and Clostridium [233]. Most dietary proteins are broken down by gastric enzymes in the upper gastrointestinal tract and absorbed by the host. The remaining proteins and peptides that reach the colon are metabolised by microbial proteases and peptidases via deamination or decarboxylation reactions to generate amino acids or SCFA, which are used in proteolytic fermentation, or to build microbial cell components [234,235]. The majority of microbial protein fermentation occurs in the distal colon, after passing through the proximal colon, where carbohydrate fermentation is dominant. Amino acids cannot be absorbed through the intestinal epithelium in the colon, therefore protein fermentation end-products can accumulate. The majority of protein fermentation end-products are branched-chain amino acids, while some bacteria such as Clostridia and Fusobacteria metabolise peptides into beneficial SCFA [236,237,238,239]. Other protein metabolites include hydrogen sulphide, phenylacetate, indoles, ammonia, and *p*-cresol, an excess of which can impair epithelial barrier function [240]. However, this has only been reported to occur in individuals with low fibre and high protein diets [241], as the availability of complex polysaccharides reduces protein fermentation by the gut microbiota [242,243]. 

### 2.6. Gastrointestinal Digestion Studies with Seaweed Peptides

Aside from being metabolised into amino acids and beneficial SCFA, seaweed-derived peptides may potentially benefit the gut by enhancing the growth and proliferation of intestinal epithelial cells. 

#### Modulation of Intestinal Epithelial Cell Differentiation

Lee et al. [244] evaluated the ability of a 20 amino acid peptide extracted from the red seaweed *Porphyra yezoensis* to modulate cell differentiation in rat intestinal epithelial (IEC-6) cells. Cells were treated with the *P*. *yezoensis* peptide (PY-PE) at concentrations of 125, 250, 500, and 1000 ng/mL for 24 h. An MTS tetrazolium assay showed that the PY-PE peptide significantly (*p* < 0.05) induced cell proliferation in a dose-dependent manner. Cells treated with 1000 ng/mL PY-PE experienced the greatest increase in numbers (65%). In order to decipher the mechanism by which the peptide exerted this effect, proteins related to the insulin-like growth factor-I receptor (IGF-IR) signalling pathway were measured in the cells. Four main insulin receptor substrate (IRS) proteins are involved in the pathway: IGF-IR, IRS-1, sarcoma homology collagen (Shc), and phosphotyrosine (PY-99). These substrates are adaptor proteins that send signals to the cell nucleus [245]. Protein and mRNA expression of these substrates by the intestinal cells after treatment with PY-PE was evaluated by western blotting, and reverse transcription-polymerase chain reaction (RT-PCR) of complementary (c)DNA. After 24 h, PY-PE successfully upregulated protein and mRNA expression of the four substrates, with the 1000 ng/mL PY-PE treatment having the most significant (*p* < 0.05) effect. 

The IGF-IR pathway in turn activates the mitogen-activated protein kinase (MAPK) signalling pathway. MAPK is a kinase (phosphate transfer enzyme) that binds with threonine and serine and directs cellular responses [246]. Expression levels of three MAPK proteins were measured: extracellular signal-regulated kinase 1/2 (ERK1/2), anti-phospho-c-Jun N-terminal kinase (JNK), and anti-phospho-p38 (P38). Treatment with PY-PE did increase (*p* < 0.05) the expression of ERK1/2 in the intestinal cells in a dose dependent manner; however, the peptide had no effect on JNK or p38. The authors surmised that the peptide only affected ERK1/2 expression because it regulates cell growth and proliferation, while JNK and p38 are activated by cellular stress and inflammation.

The effect of PY-PE on the PI3K-Akt signalling pathway was also examined by measuring the intermediates p85, p110, PDK1, and p-Akt. This pathway is involved in cell proliferation and angiogenesis through serine and threonine phosphorylation [143]. Compared to the controls, protein and mRNA expression of p85, p110, PDK1 and p-Akt was increased (*p* < 0.05) in intestinal cells treated with PY-PE, dose dependently. Lastly, the p42/p44 mitogen-activated protein kinase (MAPK1) pathway was investigated. This pathway regulates the activation of transcription factors, such as activator protein-1 and its sub-proteins, c-Jun and c-Fos, which modulate cell proliferation and differentiation. Again, PY-PE treatment successfully upregulated protein and mRNA expression of c-Jun and c-Fos in a dose dependent manner. 

Due to these positive results, the authors of the study conducted further analysis with the *P*. *yezoensis* derived peptide [247]. The proliferative effect of the peptide on the epidermal growth factor receptor (EGFR) signalling pathway was investigated in IEC-6 rat intestinal epithelial cells. The EGFR signalling pathway influences cell functions such as proliferation and involves several proteins including phosphorylated (p-)EGFR, Shc, growth factor receptor-bound protein 2 (Grb2) and son of sevenless (SOS) [248]. Treatment with the peptide (125–1000 ng/mL, 24 h) increased protein and mRNA expression of p-EGFR, Shc, Grb2 and SOS in the intestinal epithelial cells. As in the previous study, the greatest increases (*p* < 0.05) were induced by the highest concentration of peptide (1000 ng/mL). 

EGFR activates the Ras/Raf-p42/p44 MAPK signalling pathway, which mediates signal transduction from the cell surface to the nucleus [249]. The *P*. *yezoensis* peptide increased expression levels of the proteins involved in this pathway: Ras, Raf, mitogen activated extracellular kinase (MEK), and p-extracellular signal-regulated kinase (ERK) compared with the untreated control cells. 

The expression of intestinal epithelial cell cycle-related proteins was also examined. After 24 h treatment with the peptide, expression levels of proteins required for cell proliferation—cyclin D1, cyclin E, Cdk2, Cdk4, Cdk6 and pRb—increased (*p* < 0.05). Conversely, the expression of two other proteins, p21 and p27, decreased following treatment with the peptide. p21 and p27 are cyclin-dependent kinase inhibitors that regulate cell-cycle arrest for the purposes of differentiation, DNA repair, and apoptosis [250]. Although they are required for cell cycle completion, their over-expression has been linked to mucosal damage and ulcerative colitis [251]. 

Finally, the effect of the *P*. *yezoensis* peptide on cell cycle progression was measured using flow cytometry during the Gap 1 (G1) phase of cell division. Treatment with the peptide (1000 ng/mL) induced increases of 47.6, 50.6, 56.8, 62.8 and 64.4% following treatment with 0, 125, 250, 500, and 1000 ng/mL of peptide, respectively, in the proportion of cells in the G1 phase. The authors concluded from the two studies that the peptide derived from *P*. *yezoensis* seaweed has potential for development as a bio-functional food which promotes the proliferation of intestinal epithelial cells.

The bioactivity of the *P*. *yezoensis* peptide was most likely due to the ability of its structure to mimic the substrates of enzymes found *in vivo*, such as the kinases in the above *P*. *yezoensis* studies. This is known as enzymatic antagonism. Peptides can inhibit the catalytic action of enzymes on their substrates in a competitive, non-competitive, or uncompetitive manner. Competitive inhibitors can mimic and compete with normal substrates, binding with the active site of the enzyme in their stead. Non-competitive inhibitors bind to allosteric sites on the enzyme, disrupting the conformational arrangement of amino acids at the active site required for activity, thus preventing the substrate from being able to bind. Uncompetitive inhibitors bind to the enzyme-substrate complex, which changes its bioactivity [252]. Several peptides of algal origin have been shown to have chemical structures with the ability to act as enzymatic antagonists [3,9,253,254,255,256].

Table 4 summarises the peptide used in each study and significant effects observed in intestinal epithelial cells *in vitro*.

### 2.7. Bioaccessibility and Bioavailability

Bioavailability may be defined as the fraction of ingested nutrient or bioactive compound that reaches the systemic circulation and is utilised by the body [257]. Numerous factors influence the bioavailability of compounds in food including the health status of the individual, age, diet, interactions with other dietary components during digestion, and intestinal and hepatic metabolism [43,258]. Bioavailability involves two different phases—bioaccessibility and bioactivity. Bioaccessibility is the quantity of the ingested compound that is released from its food matrix and is available for absorption in the intestine [259]. Bioactivity is the biological activity of a drug or food component and involves transport of the component to the target tissue, interaction with other biomolecules, biotransformation and/or metabolism, and the induction of a physiological response [260].

The oral bioaccessibility of food can be measured *in vitro* using static or dynamic digestive methods, or *ex vivo* using organ/tissue culture models. Bioavailability can be measured using an animal-free method such as the protein digestibility-corrected amino acid score for estimating *in vivo* protein digestibility [261], but is usually measured *in vivo* using animal or human models [262]. The advantages and limitations of *in vitro* versus *ex vivo* and *in vivo* methods are here outlined.

#### 2.7.1. In Vitro Bioaccessibility Methods

*In vitro* simulated digestion methods are generally used as a preliminary test to determine the oral bioaccessibility of a food-derived component as they can be conducted in a laboratory using chemicals and enzymes that mimic the environment of the stomach and intestine without the need for live animals or human participants [263]. Experimental processes for *in vitro* simulated digestion involve several incubation steps (1–3 h) of the sample at physiological temperatures (37 °C) and conditions that simulate the mammalian digestive tract [264]. Oral digestion of the homogenised food sample begins with lingual α-amylase at pH 5–7, followed by adjustment of pH to 1–3 to mimic the stomach environment and the addition of the endopeptidase, pepsin [36]. Finally, the pH is adjusted to 6–8 to mimic the small intestine and pancreatin (a combination of amylase, protease, and lipase) is added with or without bile [265].

*In vitro* methods are divided into four categories: these are solubility, dialysability, gastrointestinal models, and cell models [245]. 

##### Solubility and Dialysability

Solubility involves centrifugation of the digested sample and quantification of the nutrient of interest in the supernatant by various techniques such as atomic absorption spectrophotometry (AA), mass spectrometry and HPLC [266]. Laparra et al. [267] estimated the bioaccessibility of arsenic in *Hizikia fusiforme*, *Porphyra* and *Enteromorpha* species using an *in vitro* solubility method followed by AA. 

Dialysability was first described by Miller et al. [268] in 1981 to measure the bioaccessibility of iron by equilibrium dialysis, and has been modified to quantify the bioaccessibility of other micronutrients. After acidic pepsin digestion of the food sample, dialysis tubing of the required MW is filled with a basic buffer such as sodium bicarbonate and added to a vessel containing the sample in its acidic environment. The sodium bicarbonate diffuses out of the dialysis tubing and neutralises the acidity. Pancreatin is added to the sample and incubated. The dialysate that diffuses in through the tubing is the bioaccessible portion of the food sample, which is then removed from the vessel and quantified [268]. 

##### Static and Dynamic Gastrointestinal Models 

Gastrointestinal models can be static or dynamic. Static models are the simpler of the two methods and involve the oral, gastric, and small intestinal stages described above. The reactions are carried out in a single bioreactor or flask with stirring and pH adjustments made at each step by addition of an acid or base, usually hydrochloric acid and sodium hydroxide [260]. One of the limitations of static methods is the broad variance in results due to the diversity of reagents used worldwide, particularly digestive enzymes, that differ in activity depending upon their source, which can be human, porcine, rabbit, bacterial, or fungal [269]. Other parameters such as incubation time, pH, ionic strength, the use of phospholipid surfactants, bile salts, and sample to liquid ratio also vary from one method to another [270]. In order to address this lack of cohesion in simulated digestive methods, the European Cooperation in Science and Technology (COST) began an EU-funded Action in 2011 called INFOGEST involving scientists from 45 countries [271]. In 2014, an international consensus was reached and a standardised static *in vitro* digestion method suitable for food was published by Minekus et al. [269] based on physiologically relevant conditions that can be applied for various endpoints. The method recommends specific concentrations and conditions for each step of static *in vitro* digestion. Pepsin was determined to be the factor causing most variation, the activity determination of which was found to be improved by pH stabilisation [269]. Subsequent inter-laboratory validation studies in 2016 by Egger et al. [265] using skim milk powder as a model food found that the harmonised INFOGEST method delivered increased consistency for the comparability of *in vitro* digestion studies. Recent studies have used the INFOGEST method to evaluate the potential bioaccessibility of seaweed components such as essential minerals [272], carrageenan [273], and to assess protein digestibility [274]. Static models have the advantage of being inexpensive, easy to use, and do not require specific equipment. However, continuous mechanical agitation is not representative of complex peristaltic movements and does not replicate the dynamic processes that occur during digestion, such as continuous changes in pH and secretions or gastric emptying [275]. 

Dynamic gastrointestinal models differ from static models in that a series of chambers are used to digest the food sample connected by peristaltic pumps [276]. The temperature, pH, enzyme concentration, incubation time and agitation-rate of each chamber is controlled by a computer [264]. The first commercial dynamic gastrointestinal model was developed in 1995 by Minekus et al. [277] at the Netherlands Organisation for Applied Scientific Research (Toegepast Natuurwetenschappelijk Onderzoek (TNO)) called the TNO Gastro-Intestinal Model (TIM). The TIM-1 model has four compartments, representing the stomach, duodenum, jejunum, and ileum connected by peristaltic valve pumps. Bioaccessible fractions are collected by dialysis after the fourth compartment [277]. The non-bioaccessible fraction is transferred to the TIM-2 model, which has one compartment representing the large intestine. Human faecal inocula is added to study the effect of colonic fermentation on the food sample and nutrient absorption [277]. The main advantage of the TIM system is that it is a holistic *in vitro* gastrointestinal model which incorporates the large as well as the small intestine. In addition, samples can be taken at any stage of the digestive process without pausing the experiment [278]. Several studies have found that bioaccessibility results using the TIM system correlate with bioavailability of the same nutrient *in vivo*. The TIM system was used to measure the bioaccessibility of iron and phosphorus from wheat [279]; folate in folate-fortified milk products [280]; and the bran, flour, and protein aleurone layer of wheat [281] and were found to be comparable to *in vivo* data. The TIM system has been used to assess the bioaccessibility of heavy metals [282] and essential minerals [283] in seaweed. Drug bioaccessibility was assessed in a study by Blanquet et al. [284] comparing the ability of TIM-1 to measure the bioaccessibility of paracetamol and a lyophilised Lactobacillus strain with *in vivo* data. The TIM1 results were consistent with *in vivo* data, showing the value of TIM-1 as a predictive tool on biopharmaceutical behaviour. However, as with all *in vitro* methods, *in vivo* factors such as first pass effect, renal clearance, and metabolisation by intestinal epithelial are not represented [284]. 

The Institute of Food Research in Norwich, England also developed a dynamic method, published by Wickham et al. [285] in 2012, called the Dynamic Gastric Model (DGM). It was designed to simulate the discrete mechanical aspects of gastric digestion as well as the biochemical and is more complex than earlier dynamic models [276]. The masticated sample is added slowly over the course of several minutes to mimic the swallowing of food. The DGM system has several functionally distinct zones in which the masticated food bolus is processed to mimic the human stomach environment. A secretion distributer gradually introduces gastric acid and enzymes to the flexible main body around the food bolus, which is then gently kneaded. Contents then move to the antrum, where they are subjected to physiological shear and grinding forces [285]. The sample, or chyme, can be removed at this stage or further digested in the duodenal chamber with pancreatic enzymes, bile salts, lecithin and cholesterol, which is often used for gastro-resistant pharmaceutical formulations to monitor dispersal and dissolution in the duodenal phase [276]. A study by Vardakou et al. [286] compared the disintegration and dissolution capabilities of the DGM system with a standard Dissolution Apparatus USP-II using agar gel beads, and compared the results to those previously observed when the same beads were given to human volunteers [287]. The DGM system was found to be superior to the Dissolution Apparatus USP-II, and there was no significant difference between the human trial data and the DGM, indicating that it is comparable to the mechanical forces exerted by the human gastric digestion [286]. Dynamic gastrointestinal models are more representative of human gastrointestinal digestion because they simulate the changing physicochemical conditions and peristaltic forces of the gastrointestinal tract; however, they are more costly and have lower throughput than static models [264]. 

Although models concerning digestion and bioaccessibility determination of food bioactives are commonly used in research today, along with colonic digestion methods, they are not always accurate or fully representative of bioactive digestion. This is because every gut has a unique microbiome that cannot currently be replicated in *in vitro* simulated models. In addition, the gut proteome plays a role in the products available for uptake. However, *in vitro* simulated models do provide a useful guide concerning the breakdown of foods/food bioactives by enzymes in the stomach and gut. Further development of *in vitro* static and dynamic models is required to give a true representation of how the microbiome and proteome of the gut impact digestion of seaweed and food bioactives. Comparisons between static, dynamic, colonic and animal studies using pigs are necessary to improve these models [225].

##### Cell Models

The fourth category of *in vitro* methods is the cell culture model. *In vitro*-differentiated human and other mammalian epithelial cell monolayers that are representative of intestinal epithelial cells can be used to mimic the ability of food components to be absorbed, and actively or passively transported and assimilated across the intestinal epithelium [288]. Cell lines commonly used for bioaccessibility studies include Caco-2, HT-29 [289], GLUTag, murine STC-1, human NCI-H716, [290], and porcine IPEC-J2 [291]. The Caco-2 cell line is a human colon carcinoma cell line which has been extensively used in gastrointestinal studies due to its spontaneous differentiation forming a monolayer of cells, which express several morphological and functional characteristics of the mature enterocyte [292]. Glahn et al. [293] expanded upon the earlier *in vitro* membrane diffusion method described by Miller et al. [268] by developing a model for assessing bioaccessibility using Caco-2 cells to measure nutrient uptake after simulated peptic and intestinal digestion of casein and various meats. The method was designed to measure iron uptake by cells but can be applied to other micronutrients. The Glahn method overcomes the issue of damage to Caco-2 cells by digestive enzymes. Normally, if a food sample that had been digested in pepsin and pancreatin were added to the media in which cells were growing, the enzymes could digest the protein structure of the cells. The Glahn method utilises a 12,000–14,000 MW cut-off dialysis membrane to allow iron (or other nutrient of interest) to diffuse through onto the cells, while the larger enzyme molecules are held back. The iron that is absorbed by the cells can then be measured. The results using this method parallel human *in vivo* absorption studies [293]. It has been used recently by Trigo et al. [294] to determine the bioaccessibility of seaweed bioactives. Flores et al. [295] and Domínguez-González et al. [296] also used the Glahn cell culture method to assess the bioavailability of iron and iodine from seaweeds. The lack of mucus production by Caco-2 cells can be a disadvantage for some studies, but may be overcome by co-culturing with a human mucus-producing cell line, such as HT29-MTX to more closely resemble *in vivo* conditions [288].

In summary, advantages of *in vitro* methods over *ex vivo* and *in vivo* include their low cost, large-scale capacity, high-throughput, and obviation of the need for human volunteers or animal testing, which is more ethical. The major limitation is the absence of the true physiological conditions of the human digestive tract such as peristalsis, phase I/II metabolism, bio-distribution, and renal excretion [262,297]. *In vitro* methods also do not fully reflect the conditions that affect digestibility *in vivo* such as interaction of the food sample with other macro- and micro-nutrients, fibre, anti-nutritional components such as phytic acid and lectins, gastric enzyme specificity, and the different absorptive capacities at each stage of the gastrointestinal tract [270,298,299,300]. *In vitro* methods offer a good preliminary measure of bioaccessibility, but bioavailability, which has a physiological or metabolic endpoint, cannot be fully quantified by *in vitro* methods [263,266].

#### 2.7.2. Ex Vivo Bioavailability Methods

*Ex vivo* organ or tissue models are also used to measure the oral bioavailability of bioactive food components. *Ex vivo* methods use living functional tissues or organs taken from an organism and maintain it in its natural physiological state [301]. The concept was first developed by Ussing [302] in 1946 to measure the active transport of sodium chloride ions in solution across frog skin. This was further developed into the Ussing chamber model, which quantifies the transport of ions, nutrients, or drugs across any epithelial tissue by measuring the potential or voltage difference that is produced as the sample diffuses in solution from one side of the epithelium to the other [303]. For oral bioavailability studies, the required mammalian intestinal mucosal tissue (from duodenum to colon) is mounted between two small chambers of buffered Ringer solution. The compound of interest, along with isotopic tracers, is added to the chamber on the lumenal (apical) side of the epithelium. To mimic haemoglobin delivery by arterial blood, levels of oxygen (95%) and carbon dioxide (5%) are maintained [304]. The active transport of the compound of interest by the epithelial cells from lumenal to mucosal side is measured by voltage difference. Interference by passive transport forces such as osmotic and electrochemical gradients is cancelled out by passing an electrical current of zero potential through the epithelium [303]. Advantages of the Ussing chamber model are its precision in measuring the electrical and transport parameters of intact epithelium, and the ability to study any type of intestinal epithelium, as well as others such as the placental barrier [275]. Its main limitations include relatively low-throughput, extensive preparation, short viability (150 min), and limited range of measurements that do not fully describe the complex physiological system of the intestinal mucosa [305].

An intestinal segment model was developed to obtain a higher throughput *ex vivo* screening system compared to the Ussing chamber model [306]. The intestinal segment model was first described in 1954 by Agar et al. [307] to measure the uptake of histidine by rat intestinal segments. The intestinal segment model measures the absorption of compounds into the intestinal cells rather than their transport through the epithelium [308]. It also involves the use of numerous sections of epithelial tissue which are cut from the original and placed in physiologically balanced solution instead of being mounted, as in the Ussing technique [307]. The porcine *ex vivo* intestinal segment model is most commonly used due to the physiological resemblance of human and pig intestines [309]. Small circles of tissue segments are punched out and incubated in buffer in 24-wells plates with the test compound. After incubation, the quantity of the test compound absorbed by the intestinal segment is quantified [310]. The intestinal segment model has advantages over the Ussing chamber model in that it is less labour intensive and has a significantly higher throughput [310].

The advantage of *ex vivo* organ models, in general, over single cell lines is that they are a multi-cell system and therefore more representative of intestinal epithelial behaviour in terms of food absorption [311,312]. Compared to *in vivo* studies, *ex vivo* organ models remove the need for human participants. Limitations of *ex vivo* organ models include the lack of inclusion of gut microbial influence and time constraints. The epithelial intestinal tissue must be excised from the animal within ~5 min of sacrifice, and the viability of intestinal tissues once the experiment begins is only ~150 min and therefore not suitable for many oral bioavailability studies that require more time [313]. The intestinal segment model has the added disadvantage of no distinction between the apical and basolateral side of the epithelium in the way that the mounted Ussing model does, as the segments are completely submerged in the same solution on both sides [314].

#### 2.7.3. In Vitro Fermentation Models

*In vitro* fermentation models allow the impact of gut microbial populations on food bioaccessibility and bioactivity to be studied without using invasive human or animal methods. Batch or dynamic fermentation models can be used [315]. Batch fermentation models entail the use of a sealed vessel under anaerobic conditions containing the food sample or extract of interest in sterile media to which is added either a pure, or mixed, bacterial culture or faecal slurry, fermented for ~2 to 24 h [316]. The advantage of batch models is that they are simple to set up and inexpensive, however, since it is a static sealed model, fermentation products such as SCFA can accumulate, and there is a finite amount of substrate available for the bacteria, all of which can affect the fermentation environment [316]. Dynamic multistage models can be used to overcome this issue. In 1988, Gibson et al. [317] first described a three-stage continuous culture system with a mixed human faecal inocula fermented over 120 days that represented the environment of the proximal, transverse and distal colon. Since the 1980s, more sophisticated, computerised dynamic models have been developed including the TIM-2 (previously discussed), The Simulator of the Human Intestinal Microbial Ecosystem (SHIME^®^) and SIMulator of the GastroIntestinal tract (SIMGI). 

The SHIME model is a 5-step multi-chamber bioreactor developed by Molly et al. [318] in 1993 that simulates the entire digestive tract from stomach to colon. The SHIME system involves allowing the microbial inoculum acclimate for 14 to 20 days to produce an environment that is representative of the *in vivo* colon in terms of bacterial populations and SCFA production [319]. Two advanced models have been developed from the original SHIME system—TWIN-SHIME and M-SHIME. Possemiers et al. [320] devised the first TWIN-SHIME model, which involves running two parallel SHIME reactors, making it possible to assess the impact of different diets or antibiotics on the same gut microbiota, as well as the metabolism and bioaccessibility of nutrients, and the pre- and probiotic effect of selected foods or microorganisms. Van den Abbeele et al. [321] incorporated mucin-covered microcosms in the M-SHIME model to create a more realistic microbial community of mucosal microbes such as *Lactobacillus mucosae* and *Pediococcus acidilactici* that are normally present on the gut epithelium. The SHIME model was used by Marzorati et al. [322] to investigate the potential of fucoidan to modulate a gut bacterial community, and by Fu et al. [323] and Calatayud et al. [324] to evaluate the effect of gut microbiota on the bioaccessibility of arsenic from the seaweeds *Hizikia fusiforme* and nori.

Advantages of SHIME include realistic representation of the upper and lower digestive tracts rather than the colon alone; long-term stability of the microbiome, which can be assessed as it adapts; option to set the model to parameters found in diverse groups such as humans, animals, diseased, healthy, elderly, or infants (Baby-SHIME) [325]; parallel comparison of alternate treatments (TWIN-SHIME); and ability to create a luminal or a mucosal microbiome (M-SHIME) [318,326,327]. Limitations of the SHIME model include a lack of realistic peristalsis, expensive set-up costs, and absence of a dialysis component and mucosal cells (in the original model) [326].

The SIMGI multicompartmental dynamic model is another five-chamber system that represents the entire human intestinal tract, developed by Barroso et al. [328] in 2015. It differs from other dynamic models in that the contents of the stomach chamber are mixed by peristaltic movements. Two rigid outer chambers surround an inner unit with flexible silicone walls. Alternating the pressure of the water flow between the outer chambers and inner unit creates a realistic simulation of gastric peristalsis [329]. The SIMGI system has the same advantages and limitations as the original SHIME model. However, SIMGI has the unique advantage of the inclusion of simulated peristalsis that is not found in SHIME or any other dynamic models, creating a more mechanically realistic stomach environment [329]. 

Overall, the advantage that all dynamic models have over static models is that they more closely represent the human gut because pH and nutrient availability within each chamber are controlled throughout the fermentation process, which also allows for much longer experiments than static batch models [330]. Dynamic models have good experimental stability and reproducibility [331]. Samples can be taken from each chamber during fermentation to assess changes in bacterial populations and their metabolites, and the ethical constraints that limit *in vivo* trials are absent [332]. Limitations of dynamic models include the lack of intestinal epithelial and immune cells in some systems; lack of host-microbe interplay [333]; no feed-back mechanisms in the system; and the use of parameters such as pH, redox potential, and transit time based on healthy individuals which may not be representative of many groups [334]. 

#### 2.7.4. In Vivo Bioavailability Methods

The most accurate method for measuring the bioavailability of a food component is *in vivo* evaluation [335]. *In vitro* and *ex vivo* methods provide very useful data on bioaccessibility, and to a certain extent, bioavailability if cell models are used but they can never fully express the digestive fate of a food component in a living person or animal. This is primarily due to the complex metabolism that occurs during absorption, where food metabolites reaching the blood system may be different from the original compounds [336]. Bioavailability involves the phases of liberation, absorption, metabolism, tissue distribution, bioactivity, and elimination [257]. Balance studies can be used to measure the oral bioavailability of a nutrient by the amount that is eliminated. This entails the collection of all urine and stools after giving a known amount of the nutrient to test subjects over several days or weeks [337]. Balance studies provide accuracy, but are laborious and more suited to laboratory animal models than human subjects [338]. Tissue distribution studies also provide bioavailability data on the extent of absorption, but are almost exclusively conducted on animals due to the invasive nature of the procedure [339,340]. In human *in vivo* studies, the oral bioavailability of a bioactive food component is most commonly measured by analysis of its metabolites in blood plasma and/or urine after a single dose, or controlled long-term consumption [336,341]. These are the methods used in the seaweed bioavailability studies discussed in the following sections.

Although *in vivo* studies are considered the gold standard for assessing the oral bioavailability of food components [263] some disadvantages exist. Compared to *in vitro* and *ex vivo* models, obtaining ethical approval for *in vivo* studies is far more difficult due to the potential harm that may be caused to animal or human participants, and in many cases, the necessary sacrifice of animal subjects [43]. *In vivo* studies are generally more expensive and time-consuming than other methods [342] and are not suitable for high-throughput screening of bioavailability [343]. It is more difficult to control all variables *in vivo* because of naturally occurring differences in living organisms, which can affect the reliability of results [344]. *In vivo* trials involving small cohorts may not be reflective of the bioavailability of a nutrient in the wider population [345]. 

However, these limitations are ultimately outweighed by the advantages. *In vivo* studies reflect the complete effect of digestion, first pass metabolism, Phase I/II biotransformation, host microbiota, and fermentation on an orally consumed nutrient [43,257]. In addition, *in vivo* studies show the impact of the nutrient on the body as a whole, rather than in one localised area or on one particular biological process [342]. Data from *in vivo* studies is more clinically relevant and any side-effects induced by the consumed sample can be observed [262,346]. Although gaps exist in *in vitro* methods of measuring digestion and bioavailability compared to animal models, *in vitro* studies still provide very relevant and useful data regarding bioaccessibility. 

Table 5 summarises the advantages and limitations of each gastrointestinal digestion model system.

A number of recent studies have evaluated the bioaccessibility of seaweed-derived polysaccharides, polyphenols, and peptides after ingestion in human and animal subjects and are discussed below.

#### 2.7.5. Bioaccessibility of Seaweed Polysaccharides

Gueven et al. [347] showed that a single dose of orally ingested fucoidan is sufficient to affect the expression of genes related to immunity, inflammation, cancer, and neurological function. A placebo-controlled double-blind study was performed in nine healthy, male volunteers (age 25–65 years-old). Fucoidan (85.1% pure, MW 47.7 kDa) was water-extracted from *U. pinnatifida* harvested in Tasmania. A capsule containing 1 g seaweed extract (851 mg fucoidan) or a cellulose placebo was administered. Blood was taken immediately before and 24 h after ingestion. 754 micro RNA (miRNA) strands were isolated and analysed as biomarkers of physiological function. Fucoidan ingestion was found to affect 53 miRNAs. Fifteen were upregulated and 38 downregulated. Only one upregulated and five downregulated miRNAs were common to both the placebo and fucoidan groups. The pathways and processes affected by the identified miRNAs are associated with cell surface receptor signalling, the enhancement of brain-derived neurotrophic factor, epidermal growth factor receptor, insulin receptors, and the associated MAPK downstream signalling.

Ikeda-Ohtsubo et al. [348] evaluated the *in vivo* modulatory effects of fucoidan on the gut microbiota in an animal model. Fucoidan (>95% pure, MW 49.8 kDa) was extracted from *Cladosiphon okamuranus* (Okinawa mozuku) harvested in Japan. Adult zebrafish had their food supplemented (1:1) with fucoidan for 3 weeks. The presence of pro- and anti-inflammatory cytokines was determined by quantitative (q)PCR. Then, 16S rRNA sequencing was used to analyse changes in the microbiota. There was a significant decrease in expression levels of the pro-inflammatory cytokine IL-1β in the fucoidan-fed zebrafish compared to the control. In terms of beneficial changes to the microbiota, fucoidan feeding significantly enhanced the diversity and composition of intestinal bacterial. Bacteria of the families Rhizobiaceae (genus Shinella) and Comamonadaceae (genus unclassified) became dominant at the expense of *E*. *coli*-related Enterobacteriaceae. Intestinal Enterobacteriaceae have been reported to have pro-inflammatory effects [349]. The reduction in Enterobacteriaceae after fucoidan supplementation may have been responsible for the downregulation of the pro-inflammatory cytokine IL-1β. This illustrates the potential modulatory role of seaweed polysaccharides in the diet–microbiota–host interplay.

Fucoidan extracted from Japanese Okinawa mozuku was also shown to be bioaccessible to rats fed 2% fucoidan-supplemented food for 8 weeks [350]. Immunohistochemical staining revealed that fucoidan had been absorbed across the intestinal epithelium and taken up by intestinal macrophages and hepatic Kupffer cells. The same research group went on to investigate factors concerning the absorption of the Okinawa mozuku-derived fucoidan in a cross-sectional human study (*n* = 396) by Kadena et al. [351]. Okinawa mozuku is a species of brown seaweed endemic to a small group of islands south of the Japanese mainland called the Okinawa prefecture or region. Of the study population, 68% were native to Okinawa, while 32% were from other regions of Japan. Participants (227 male, 169 female, age 20 to >70 years-old) were administered a drink of 3.75 g mozuku extract (containing 3 g pure fucoidan) in 200 mL purified water. The fucoidan had an average MW of 73.4 kDa and a composition of 51.2% L-fucose, 14.4% uronic acid, and 18.8% sulphate. Participants refrained from consuming seaweed or fucoidan supplements the day before and throughout the day of trial. Urine samples were collected before administration and 3, 6, and 9 h after. The presence of fucoidan was measured using a purpose-designed ELISA [352]. The assay antibody was specific to fucoidan and did not react with other sulphated polysaccharides. Fucoidan concentration was expressed as a corrected urinary creatinine value (µg/gCr) as fucoidan was calculated to be equivalent to one eighth of urinary excreted creatinine. 

The results showed that intestinal absorption of Okinawa mozuku-derived fucoidan occurred in 97% of study participants (385 of 396). There was a highly significant difference (*p <* 0.01) in fucoidan absorption in native Okinawa participants compared to those from other regions. Eight of the 11 participants who did not excrete fucoidan lived outside Okinawa. After 9 h, the total mean urinary fucoidan content of native Okinawa participants (332.3 μg/gCr) was 38.4% greater (*p <* 0.01) than those from other regions (240.1 μg/gCr). Of the group, 87.5% that excreted the highest levels fucoidan (>1200 μg/gCr) were native to Okinawa. By age bracket, participants in their 40 s had the greatest mean urinary fucoidan value (392.8 μg/gCr). The authors hypothesised that the gut bacteria of native Okinawa participants may have acquired genes from marine bacteria that produce the digestive enzyme fucoidanase. This horizontal transfer of genes from ocean-dwelling bacteria that normally colonise and feed on seaweed has previously been reported in populations that have consumed seaweed for thousands of years [353,354,355,356,357].

#### 2.7.6. Bioaccessibilty of Seaweed Polyphenols 

Human clinical studies on the bioaccessibility of seaweed polyphenols are limited to brown species, and phlorotannins in particular. Table 6 summarises the polyphenol used in each study and the impact of digestion on their bioaccessibilty.

These studies, along with others that have assessed the bioaccessibilty of polyphenols from terrestrial plants, have a commonality in that oral bioaccessibilty of polyphenols in some individuals is poor. There are a number of reasons for this, such as host-related factors. These can be systemic factors including age, gender, genetics, and existing health disorders; or intestinal factors, such as gastric enzyme activity, intestinal transit time, and gut microflora composition intestinal factors such as gastric enzyme activity, intestinal transit time, and gut microflora composition [359]. The food matrix in which polyphenols are consumed and interactions with macronutrients impact bioaccessibility. In the digestive tract, the amine group of proteins can form irreversible covalent bonds with the carboxylic group of polyphenols [360], which interferes with the ability of digestive enzymes to catabolise them. Since enzymes are proteins, polyphenols also interact with their amine groups, further inhibiting digestion. [361,362]. The capacity for polyphenols to bind with proteins increases with their molecular weight. Some seaweed polyphenols, such as phlorotannins, have a MW of up to 100 kDa [363], making them suitable candidates for multiple protein-polyphenol interactions. 

Lipids have been shown to enhance polyphenol bioaccessibilty. Hydrophobic interactions between lipids and polyphenols have a protective effect and increase the stability of polyphenols during digestion [364]. Complexing with lipids can also increase the accumulation of polyphenols in the liver, which acts as a slow-release reservoir that prolongs their residence time in the blood [365]. Polysaccharides, in the form of dietary fibre, can bind with polyphenols. The hydroxyl groups of polyphenols form hydrogen bonds with the oxygen atoms of polysaccharide glycosidic linkages [366] or covalent bonds, such as esters [367]. While this reduces the ability of gastric enzymes to make them bioaccessible in the upper gastrointestinal tract, polyphenols can be released from their non-digestible polysaccharide complex in the colon through the action of gut microbial digestive enzymes [368]. In fact, polysaccharides such as alginate have been used to encapsulate polyphenols, delaying their release until they reach the colon [369].

Despite their low oral bioaccessibility, the biological activity of polyphenols is generally found to be high, leading to a low bioaccessibility/high bioactivity paradox. This is most likely due to the biotransformation of polyphenols in the liver and enterocytes mediated by phase I cytochrome P450 enzymes and phase II conjugation enzymes (uridine 5’-diphospho-glucuronosyltransferase and sulphotransferase) [343]. Phase I and II biotransformation is a detoxification system that modifies compounds that the body perceives as xenobiotics for easier excretion via urine, faeces, and bile [370]. This biotransformation results in conjugated compounds with different polarity, MW, ionic form, and greater intrinsic biological effects than their parent compounds [371,372]. After compounds such as polyphenols are conjugated, they re-enter the gastrointestinal tract in bile via enterohepatic recirculation [373]. Gut bacterial enzymes, particularly β-glucuronidase, can metabolise many of these polyphenol conjugates, further modifying their chemical structure, bioactivity, and bioavailability [341]. This enterohepatic recycling prolongs the presence of polyphenols within the body. Therefore, the limited oral bioaccessibilty of seaweed polyphenols does not determine their ultimate bioactivity. The biotransformation of native polyphenols through the action of digestive enzymes and microbial fermentation produces metabolites with disparate bioaccessibilty and bioactivity.

#### 2.7.7. Bioaccessibility of Seaweed Peptides

There is a dearth of literature on the *in vivo* bioaccessibility of seaweed-derived peptides in human studies; however, some *in vivo* studies have reported the effect of digestion on their bioactivity in animal models or *in vitro*. Table 7 summarises the peptide used in each study and the impact of digestion on their bioactivity.

Despite the lack of human clinical data on the bioavailability of seaweed peptides, *in vivo* animal trials and *in vitro* simulated gastrointestinal digestion studies provide relevant information on the ability of bioactive peptides, sometimes termed cryptides, to retain their efficacy after transit through the mammalian gut [375,376]. 

## 3. Conclusions

The aetiology of many leading global chronic disorders such as inflammatory disease, immunodeficiency, metabolic syndrome and cancer has been linked to dysbiosis of the gut. *In vitro*, animal, and human studies collated in this review show that the consumption of seaweed components may have the potential to beneficially modulate the microbiota of the mammalian gut. Seaweed polysaccharides such as fucoidan, laminarin, alginate, ulvan and porphyran have shown particular efficacy as modulators of the gut by acting as prebiotics, which increase gut bacterial numbers and the production of short chain fatty acids. There are, however, many factors that can reduce the bioaccessibility and bioavailability of seaweed components. These include antagonistic or synergistic interactions with other food components; physiochemical digestibility parameters such as solubility, polarity, molecular weight, surrounding food matrix; and the impact of first-pass metabolism. However, the low bioavailability of some seaweed components can be modified by gastric, enterohepatic, and bacterial biotransformation resulting in compounds with enhanced bioactivity. Another factor that affects bioaccessibility and bioavailability is the composition of each individual’s gut microbiota, which varies broadly. This may lead to the absence of certain bacterial families required for the metabolism of seaweed components. This can be augmented by introducing bacterial strains capable of digesting them. There is a dearth of data available in the literature on human dietary intervention studies with seaweed polysaccharides, polyphenols and peptides. Although *in vitro* studies and *in vivo* animal trials are an indication of the prebiotic potential of seaweed components, they are not fully representative of how the component will be metabolised in humans. Further randomised controlled clinical trials are required in large human cohorts, with measureable endpoints, to validate any putative health effects observed in animal models, simulated digestion models, or *in vitro*. With the practice of mariculture becoming more widespread globally, seaweeds represent a sustainable source of bioactive compounds with potential to be used as modulators of the gut microbiota. 

## Figures and Tables

**Table 1:** The impact of polysaccharides on gut bacteria.

Polysaccharide	Seaweed	Extraction Method	Study Type	Statistically Significant Effects	Ref.
* (i) Crude polysaccharide-rich extract (>1 kDa) (CE) (ii) Depolymerised crude extract (>1 kDa) (DE)	*L. digitata*	(i) (CE) Hot acid and ethanol precipitation (0.1 M HCl) (ii) (DE) Fenton’s reaction with iron and hydrogen peroxide	Simulated *in vitro* colonic digestion	After 24 h fermentation, compared to cellulose control: CE increased relative abundance of *Porphyromonadaceae* (*p* = 0.043), *Lachnospiraceae* (*p* = 0.015) and *Dialister* (*p* = 0.005); and reduced *Fibrobacteraceae* (*p* = 0.026) *Streptococcaceae* (*p* = 0.025), *Ruminococcus*, (*p* = 0.027) *Streptococcus* (*p* = 0.022) and *Fibrobacter* (*p* = 0.026). DE increased *Parabacteroides* (*p* = 0.017) *Lachnospiraceae* (*p* = 0.039), *Dialister* (*p* = 0.008) and reduced *Alcaligenaceae* (a Proteobacterium) (*p* = 0.030) and *Peptostreptococcaceae Incertae Sedis* (*p* = 0.027). CE and DE increased total SCFA, acetic, propionic, and butyric acid (all *p* < 0.05) after 10, 24, 36, and 48 h. Ratio of propionate to acetate beneficially reduced by CE and DE (both *p* < 0.05) after 24, 36, and 48 h.	[147]
* Porphyran, ulvan and laminarin	Pyropia, Ulva and Laminaria	Ethanol (80%)	Simulated *in vitro* colonic digestion	After 24 h fermentation, growth of bacterial genera compared to fructooligosaccharide (FOS) control: Porphyran increased Lactobacilli (10.7%, *p* < 0.05). Ulvan increased Bacteroides (6.7%, *p* < 0.05). Laminarin increased Bifidobacteria (8.3%, *p* < 0.05) and Bacteroides (13.8%, *p* < 0.05). Negative results: no significant increase at 24 h in total SCFA, butyrate, lactate or acetate by laminarin, ulvan or porpyran compared to FOS.	[132]
* (i) Crude extract fraction (CF) (ii) Low MW fraction (LPF) (iii) High MW fraction (HPF)	*E. radiata*	(i) Enzymatic (Viscozyme-β-glucanase, hemicellulase, arabanase, xylanase) (ii and iii) Viscozyme and ethanol precipitation	Simulated *in vitro* colonic digestion	Increases (log_10_ cells/mL) after 24 h fermentation (all *p* < 0.05 compared to controls): ▪ Bacteroidetes (CF 7.36 ± 0.03, LPF 7.21 ± 0.05 and HPF 7.28 ± 0.04) greater than cellulose (6.40 ± 0.05). ▪ *Faecalibacterium prausnitzii* (CF 6.34 ± 0.05, LPF 6.42 ± 0.08) greater than inulin (6.17 ± 0.04) and cellulose (6.07 ± 0.06). ▪ *Clostridium coccoides* (CF 8.29 ± 0.03, LPF 8.56 ± 0.06) greater than inulin (7.57 ± 0.06) and cellulose (7.40 ± 0.05) ▪ *Escherichia coli* (CF 7.16 ± 0.04, LPF 7.31 ± 0.05 and HPF 6.96 ± 0.04) greater than cellulose (6.81± 0.03) ▪ Bifidobacteria (LPF 7.11 ± 0.12) greater than cellulose (6.34 ± 0.06) ▪ Lactobacilli (LPF 6.56 ± 0.05) greater than inulin (6.07 ± 0.05) and cellulose (5.11 ± 0.06) SCFA production after 24 h (all *p* < 0.05): ▪ Total SCFA in CF (97.3 μmol/mL), LPF (89.0 μmol/mL) greater than inulin positive control. HPF (68.9 μmol/mL) greater than cellulose (39.7 μmol/mL) but ~20% lower than inulin. ▪ Acetic acid HPF (40.8 μmol/mL) > cellulose ▪ Propionic acid CF (54.6 μmol/mL) > inulin and cellulose ▪ Butyric acid LPF (17.3 μmol/mL) > inulin and cellulose Ratio of Firmicutes to Bacteroidetes beneficially lowered: HPF (1.08 ± 0.008), CF (1.14 ± 0.001) and LPF (1.18 ± 0.006) compared to cellulose (1.22 ± 0.004). Ratio of propionic acid to acetic acid beneficially reduced: 0.47 ± 0.04 (CF), 0.62 ± 0.06 (LPF) and 2.15 ± 0.06 (HPF) compared to 4.08 ± 0.18 (inulin) and 5.73 ± 0.13 (cellulose).	[10]
* (i) Low MW polysaccharide (LMW) (primarily laminarin) (ii) High MW polysaccharide acidic water extract (HMW) (primarily fucoidan and alginate) (iii) High MW polysaccharide water and ethanol precipitate (HMWW) (primarily fucoidan and alginate)	*E. radiata*	(i) Enzymatic (cellulase) (ii) Acidic water (pH 4.5) (iii) Water and ethanol precipitation	Simulated *in vitro* colonic digestion	24 h post fermentation (all differences *p* < 0.05): (i) LMW increased Bifidobacteria from 5.51 ± 0.15 log_10_ cells/mL (in cellulose fermented control) to 6.55 ± 0.08 log_10_ cells/mL; Lactobacillus from 4.73 ± 0.13 (cellulose) to 5.28 ± 0.19 log_10_ cells/mL and Bacteroidetes from 5.09 ± 0.06 (cellulose) to 6.02 ± 0.09 log_10_ cells/mL. Negative results: no significant increase by LMW on populations of *F. prausnitzii, Clostridium leptum*, *Ruminococcus bromii*, *E. coli* or *Enterococcus*. (ii) HMW increased *C. coccoides* from 5.74 ± 0.75 (cellulose) to 7.07 ± 0.04 log_10_ cells/mL, *E. coli* from 6.09 ± 0.41 (cellulose) to 7.52 ± 0.07 log_10_ cells/mL and *Enterococcus* from 5.02 ± 0.31 (cellulose) to 6.63 ± 0.11 log_10_ cells/mL. Negative results: no significant increase by HMW in any other bacterial populations. (iii) HMWW increased *E. coli* from 6.09 ± 0.41 (cellulose) to 7.01 ± 0.17 log_10_ cells/mL and *Enterococcus* from 5.02 ± 0.31 (cellulose) to 5.80 ± 0.33 log_10_ cells/mL. HMWW also had a negative effect on several bacterial populations—Bifidobacteria reduced from 5.51 ± 0.15 (cellulose) to 3.21 ± 0.61 log_10_ cells/mL, Bacteroidetes from 5.09 ± 0.06 (cellulose) to 4.08 ± 0.12 log_10_ cells/mL, Lactobacillus 4.73 ± 0.13 log_10_ cells/mL (cellulose) to not detected (ND), *C. coccoides* from 5.74 ± 0.75 log_10_ cells/mL (cellulose) to ND, *C. leptum* from 6.23 ± 0.28 log_10_ cells/mL (cellulose) to ND and *R. bromii* from 6.20 ± 0.06 (cellulose) to 4.87 ± 0.29 log_10_ cells/mL. SCFA increases in seaweed ferments vs. cellulose control after 24 h (all *p* < 0.05): LMW ▪ Total SCFA 63.42 ± 1.76 vs. 18.59 ± 0.14 μmol/mL ▪ Acetic acid 22.81 ± 0.91 vs. 9.09 ± 0.07 μmol/mL ▪ Propionic acid 29.61 ± 2.60 vs. 3.24 ± 0.04 μmol/mL ▪ Butyric acid 9.22 ± 1.38 vs. 2.02 ± 0.03 μmol/mL 2. HMW ▪ Total SCFA 62.86 ± 0.20 vs. 18.59 ± 0.14 μmol/mL ▪ Acetic acid 20.59 ± 0.21 vs. 9.09 ± 0.07 μmol/mL ▪ Propionic acid 36.79 ± 0.57 vs. 36.79 ± 0.57 μmol/mL ▪ Butyric acid 4.27 ± 0.48 vs. 2.02 ± 0.03 μmol/mL 3. HMWW ▪ Total SCFA 50.70 ± 1.10 vs. 18.59 ± 0.14 μmol/mL ▪ Acetic acid 27.05 ± 0.58 vs. 9.09 ± 0.07 μmol/mL ▪ Propionic acid 18.20 ± 0.38 vs. 3.24 ± 0.04 μmol/mL ▪ Butyric acid—no significant increase	[148]
** (i) Polysaccharide fraction (PF) (primarily fucoidan and alginate) (ii) Whole seaweed (WS)	*E. radiata*	(i) Enzymatic (Viscozyme) (ii) Whole dried *E. radiata*	*In vivo* trial with healthy Sprague-Dawley rats (7 d, 5% PF or 5% WS added to feed)	After 7 days supplementation (all differences *p* < 0.05): Reduction in potentially pathogenic Enterococci in WS group (6.04 ± 0.09 log_10_ cells/mL) vs. control (5.59 ± 0.08 log_10_ cells/mL) Increase in butyrate-producing *F. prausnitzii* in PF group (5.32 ± 0.11 log_10_ cells/mL) vs. control (4.87 ± 0.11 log_10_ cells/mL) 2-fold increase in caecal digesta mass 1.36 ± 0.17 (PF) vs. 0.60 ± 0.06 g/100 g BM (control) Putrefactive microbial products reduced (all values µg/g caecal digesta): phenol in WS (0.36 ± 0.03) and PF (0.49 ± 0.02) vs. control (2.91 ± 0.70) *p*-cresol in WS (0.47 ± 0.05) SCFA increase in WS (213.25 ± 14.40 µmol) and PF (208.59 ± 23.32 µmol) vs. control (159.96 ± 13.10 µmol)Negative results: – No significant *p*-cresol decrease in PF fed rats (19.34 ± 5.14) vs. control (25.18 ± 6.18 µg/g caecal digesta)	[149]
* (i) conventional chemical extraction (CCE) (11.9% fucoidan) (ii) microwave-assisted extraction (MAE) (5.71% fucoidan) (iii) ultrasound-assisted extraction (UAE) (4.56% fucoidan) (iv) enzyme-assisted extraction (EAE) (3.89% fucoidan)	*A. nodosum*	(i, ii, and iii) Ethanol followed by acidic water (0.01 M HCl) (iv) Cellulase, acetate buffer (pH 4.5)	*L. casei* and *L. delbrueckii* ssp. *bulgaricus* broth cultures, 3.75% (*v*/*v*). *A. nodosum* extracts added at 0.1%, 0.3% and 0.5% (*w*/*v*)	All differences *p* < 0.05 compared to non-supplemented control medium: Increase in *L. delbrueckii* ssp. *bulgaricus* by CCE, MAE, UAE and EAE at 0.1%, 0.3% and 0.5%. Increase (24.5%) in *L*. *casei* only by MAE at 0.5% inclusion. Negative results: – No significant increase in *L. casei* by CCE, UAE or EAE vs. non-supplemented media.	[87]
* Crude sulphated polysaccharide (716 kDa) (90% galactose, 9.07% sulphate)	*C. pilulifera*	Acidic extraction (0.0.1 M HCl) and ethanol precipitation	Simulated *in vitro* saliva, gastric, small intestinal and colonic digestion	After 24 h, all differences *p* < 0.05 compared to inulin control: Increase in Bacteroides, Parabacteroides, Megamonas and Veillonella. Increase in total SCFA (22.17 ± 0.82 mmol/L) vs. control (16.17 mmol/L ± 0.39). Negative results: – No significant increase in butyrate, lactate, iso-butyrate, valerate or iso-valerate in seaweed polysaccharide supplemented ferments.	[150]
* (i) Polysaccharides (SJP) (138 kDa) (Fucose:galactose:glucuronic acid:mannose, molar ratio of 4.1:3.6:1.2: 1.0). (ii) Oligosaccharides (SJO)	*S. japonica*	(i) Methanol, dichloromethane, water and ethanol (ii) Methanol, dichloromethane, water and ethanol, followed by 0.6 M HCl	Simulated *in vitro* colonic digestion	After 24 h, all differences *p* < 0.05 compared to FOS control Increase in beneficial Bacteroidetes and decrease in Proteobacteria (SJP and SJO). Increased ratio of Bacteroidetes to Firmicutes (SJP and SJO).	[91]
** Crude sulphated polysaccharide (SP) (28.807 kDa) (Galactose (59.7%), galacturonic acid (19.8%), xylose (7.1%) and sulphate (8.8%))	*G. pacificum*	Ultrasound-assisted water extraction followed by ethanol, acetone and petroleum precipitation	*In vivo* trial with lincomycin hydrochloride induced diarrhoeal mice (9 days, 75 mg SP/kg BM)	After 9 d, seaweed polysaccharide group vs. non-supplemented normal recovery group (all differences *p* < 0.05): Increase in beneficial Bacteroides, Oscillospira and Bifidobacterium. Decrease in Parabacteroides, Sutterella and AF12. Reduction in inflammatory cytokines, TNF-α, IL-1β and IL-2. Improved (lower) diarrhoea status scores, water intake, and less weight loss. Increase in total SCFA, acetate and propionate.	[151]
** Fucoidan (300 kDa) (60% fucose, 14.3% sulphate)	*C. okamuranus*	Method not specified	*In vivo* trial with Traf3 ip2-mutant psoriasis mice (fucoidan diet group n = 14, normal diet group n = 9, 63 days, 1% fucoidan added to feed)	Fucoidan group vs. cellulose control group (all differences *p* < 0.05). After 56 days: ▪ Increase (% relative abundance) in Bacteroidetes (78.2 ± 6.42 vs. 59.4 ± 9.69%), Proteobacteria (3.05 ± 0.62 vs. 1.73 ± 0.53%), and Paraprevotellaceae. ▪ Decrease in Firmicutes (16.3 ± 4.98 vs. 34.3 ± 9.05%) and TM7 Saccharibacteria (3.80 ± 0.24 vs. 1.23 ± 0.11%). ▪ After 21 days increase in mucin production in ileum and faeces ▪ After 63 days increase in IgA production in cecum+ ▪ Reduction in psoriasis area and severity index (PASI) and ethological scratch-test Negative results: – Decreases in Deferribacteres and Actinobacteria after 56 days were not significant	[89]
** Laminarin and fucoidan (10% laminarin,8% fucoidan and 82% ash)	*Laminaria hyperborea*	Method not specified	*In vivo* trial (10 pregnant sows/treatment) (10 g/days seaweed extract from day 107 of gestation until weaning (day 26)) and *ex vivo* lipopolysaccharide (LPS) immunological challenge	Compared with non-supplemented group, seaweed extract supplemented (SWE) sows had: ▪ Greater colostrum IgA (*p* < 0.01) and IgG (*p* = 0.062) ▪ Decreased faecal Enterobacteriaceae populations at parturition (*p* < 0.05) ▪ Reduced faecal *Enterobacteriaceae* on expected farrowing date (7.26 vs. 8.60 log_10_ CFU/g, pooled SEM 0.463, *p* < 0.05) LPS challenge increased pro-inflammatory cytokines IL-1α and IL-6 (*p* < 0.01) in ileal tissue and tumor necrosis factor (TNF)-α in colonic (*p* < 0.01) tissue Piglets suckling SWE sows had: ▪ Greater TNF-α after *ex vivo* LPS challenge (*p* < 0.05) ▪ Increased serum IgG (*p* < 0.05) on day 14 ▪ Decreased colonic *E. coli* population (*p* < 0.01) at weaning ▪ Greater Lactobacilli: *E.coli* ratio (*p* < 0.05) Negative results: – No increase in faecal volatile fatty concentrations in SWE sows – SWE diet had no effect on TNF-α mRNA expression in unchallenged sow ileal tissue – Piglet birth and weaning weight, and small intestinal morphology unaffected by SWE sow diet	[101]

* = *in vitro* studies; ** = *in vivo* animal studies.

**Table 2:** The potential impact of polyphenols on the gut microbiota *in vitro* and *in vivo*, modulation of hyperglycaemia in animal models and DNA damage *in vitro*.

Polyphenol	Seaweed	Extraction Method	Study Type	Statistically Significant Effects	Ref.
* Phlorotannin enriched fraction	*E. radiata*	Ethanol (90%)	Simulated *in vitro* colonic digestion	Increases (all *p* < 0.05) in Bacteroidetes (6.52 ± 0.04 log_10_ cells/mL) compared to the cellulose control (6.40 ± 0.05 log_10_ cells/mL); *F. prausnitzii* (6.57 ± 0.05 log_10_ cells/mL) compared to cellulose and inulin controls (6.17 ± 0.04 and 6.07 ± 0.06 log_10_ cells/mL, respectively); *C. coccoides* (7.97 ± 0.05 log_10_ cells/mL) compared to inulin and cellulose controls (7.57 ± 0.06 and 7.40 ± 0.05 log_10_ cells/mL, respectively); and *E. coli* (8.09 ± 0.02 log_10_ cells/mL) compared to inulin and cellulose controls (6.81 ± 0.03 and 6.94 ± 0.03 log_10_ cells/mL, respectively).	[10]
** Polyphenols (3 kDa) (luteolin-6-c-glucoside, regiolone, neoeriocitrin and estr-5(10)-ene-3,17-diol)	*E. prolifera*	Ultrasound assisted ethanol extraction (55%) and ultrafiltration (3 kDa)	*In vivo* trial with diabetic mice (4 weeks, 300 mg polyphenol extract/kg BM/day)	Reduction after 14 days (*p <* 0.05) in mean BM of *E. prolifera*-fed diabetic group compared to model diabetic group. Reduction after 28 days (*p <* 0.05) in mean fasting blood glucose levels of *E. prolifera*-fed diabetic group and glucose tolerance increased (*p <* 0.05) compared to the model diabetic group. Increase in Alistipes (*p <* 0.05) in *E. prolifera*-fed diabetic group compared to model diabetic group. Hypoglycaemic effect via increase (*p <* 0.01) in phosphatidylinositol 3-kinase and suppression (*p <* 0.05) of c-Jun N-terminal kinase in *E. prolifera*-fed diabetic group livers compared to model diabetic group.	[170]
** Polyphenol-rich fraction (primarily phlorotannins, phenolic acids and gallocatechin derivatives)	*L. trabeculata*	Microwave assisted methanol extraction, solvent fractionation and macroporous resin adsorption separation	*In vivo* trial with diabetic rats (4 weeks, 200 mg/day phlorotannin extract/kg BM)	Increase in genera of the phylum Bacteroidetes in the PE group compared to the DC group: Odoribacter (*p* < 0.008), Muribaculum (*p <* 0.005), Alistipes (*p <* 0.006), Lachnospiraceae (*p <* 0.015) and Parabacteroides (*p <* 0.022). Decrease in Proteobacteria, and ratio of Firmicutes to Bacteroidetes (*p* < 0.05 PE vs. DC group). Increase in total SCFA (491.31 ± 10.39 (DC), 1276.34 ± 16.86 μg/g (PE) (*p* < 0.01)), acetic acid (377.77 ± 3.46 (DC), 1202.49 ± 11.55 μg/g (PE) (*p* < 0.01)) and butyric acid (10.18 ± 0.58 (DC), 39.77 ± 1.85 μg/g (PE) (*p* < 0.01)). Reduction in the PE group versus the DC group in: fasting blood glucose (10.55 ± 0.94 vs. 13.99 ± 0.87 mmol/L (*p* < 0.05)), serum insulin (14.69 ± 0.11 vs. 17.70 ± 0.22 mU/L (*p* < 0.01)), HOMA-IR insulin resistance value (6.89 ± 0.42 vs. 11.01 ± 0.98 (*p* < 0.01)), total cholesterol (4.92 ± 0.14 vs. 5.64 ± 0.16 mmol/L (*p* < 0.01)), triglycerides (0.99 ± 0.04 vs. 1.43 ± 0.10 mmol/L (*p* < 0.01)), LDL cholesterol (0.68 ± 0.03 vs. 1.06 ± 0.06 (*p* < 0.01)), glycated serum protein (2.15 ± 0.16 vs. 2.74 ± 0.15 (*p* < 0.01)) and non-esterified fatty acids (1.86 ± 0.05 vs. 2.02 ± 0.11 mmol/L (*p* < 0.05)).	[210]
(i) * Phlorotannin (HMW > 10 kDa) (ii) Phlorotannin (LMW 1–10 kDa)	*A*. *nodosum*	Ethanol	(a) *In vitro* gastrointestinal digestion and colonic fermentation (b) H_2_O_2_ induced DNA damage in HT-29 colon cancer cells	(a) Reduction in MW of phlorotannins (89.9% HMW, 62.0% LMW) by colonic fermentation, compared to enzymatic gastric digestion (5.4% HMW, 52.8% LMW), suggesting phlorotannins may potentially be metabolised by human gut bacteria. (b) Compared to the control, HMW and LMW phlorotannin extracts at a concentration of 500 μg/mL inhibited (*p <* 0.01) HT-29 colon cancer cell proliferation (number of cells by division), HMW inhibited (*p <* 0.05) HT-29 cell growth (mass accumulation) at concentrations of 250 and 500 μg/mL. H_2_O_2_ induced DNA damage in HT-29 cells reduced by post-gastric digested HMW extract (*p <* 0.01) and HMW and LMW post-colonic fermented extracts (both *p <* 0.001).	[216]

* = *in vitro* studies; ** = *in vivo* animal studies

**Table 3:** Amino acid sequences of recently elucidated seaweed-derived peptides and their bioactivities *in vitro*, *in silico* or *in vivo*.

Seaweed	Extraction Method	Amino Acid Sequence	Bioactivity	Ref.
* ^†^ U. lactuca	Enzymatic (Papain), MWCO filtration, preparative RP-HPLC and *in silico* enzyme cleavage simulation	(i) Ala-Thr-Lys-Pro-Ala-Asn (ii) Ser-Gly-Ala-Ala-Ser-Ala-Ser-Gly-Ala-Ala (iii) Ala-Gly-Gly-Pro-Asn-Gln-Pro-Pro-Asn (iv) Ala-Ala-Asn-Ile-Thr-Val-Pro-Ala-Ala-Asn (v) Glu-Ala-Glu-Pro-Ala-Glu-Ala-Ala (vi) Gly-Ala-Ala-Pro-Thr-Pro-Pro-Ser-Pro-Pro-Pro-Ala-Thr-Lys-Pro-Ser-Thr-Pro-Pro-Lys-Pro-Pro-Thr (vii) Pro-Pro-Asn-Pro-Pro-Asn-Pro-Pro-Asn Amino acid sequences not defined: (a) crude seaweed protein (b) full peptide hydrolysate (c) 1 kDa-UFH (ultra-filtered hydrolysate) (d) 3 kDa-UFH (e) 10 kDa-UFH	Peptides (i) to (vii) ACE-I, DPP-IV, and enzyme 3-hydroxy-3-methyl-glutaryl-CoA reductase inhibition (*in silico* predictive activity) *In vitro* ACE-I inhibitory activity (%) (all assayed at conc. of 1mg/mL): (a) crude seaweed protein 79.87 ± 0.18% (b) full peptide hydrolysate 82.37 ± 0.05% (c) 1 kDa-UFH (ultra-filtered hysrolysate) 93.03 ± 0.87% (d) 3 kDa-UFH 86.64 ± 2.17% (e) 10 kDa-UFH 88.12 ± 0.02%	[9]
* *P. palmata*	Enzymatic (Papain)	Ile-Arg-Leu-Ile-Ile-Val-Leu-Met-Pro-Ile-Leu-Met-Ala	Renin inhibition (58.97 ± 1.26% inhibition *in vitro* at 1 mg/mL)	[217]
* *P. palmata*	Enzymatic (Protease)	(i) Ile-Leu-Ala-Pro (ii) Leu-Leu-Ala-Pro (iii) Met-Ala-Gly-Val-Asp-His-Ile	DPP-IV inhibition IC_50_ values *in vitro*: (i) 43.40 ± 1.40 μM (ii) 53.67 ± 0.82 μM (iii) 159.37 ± 13.67 μM	[218]
* *P. palmata*	Enzymatic (Papain)	Asn-Ile-Gly-Lys	PAF-AH inhibition IC_50_ value *in vitro* 2.32 ± 2.12 mM	[219]
* Porphyra (Laver—species not specified)	Enzymatic (Viscozyme, Alcalase, Neutrase, Pepsin and Trypsin)	(i) Gly-Gly-Ser-Lys (ii) Glu-Leu-Ser	α-amylase inhibition IC_50_ values *in vitro*: (i) 2.58 ± 0.08 mM (ii) 2.62 ± 0.05 mM	[220]
* *P. palmata*	Thermolysin hydrolysis	(i) Leu-Arg-Tyr (ii) Val-Tyr-Arg-Thr	ACE-I inhibition IC_50_ values *in vitro*: (i) 0.044 μM (ii) 0.14 μM	[228]
*,** *U. pinnatifida*	Enzymatic (Protease)	(i) Val-Tyr (ii) Ile-Tyr (iii) Phe-Tyr (iv) Ile-Trp (v) Ala-Trpvi) Val-Trp (vii) Leu-Trp	ACE-I inhibition IC_50_ values *in vitro*: (i) 35.2 μM (ii) 6.1 μM (iii) 42.3 μM (iv) 1.5 μM (v) 18.8 μM(vi) 3.3 μM (vii) 23.6 μM *In vivo* antihypertensive effect in spontaneously hypertensive rats (single oral dose, 1 mg/kg of BW). Blood pressure decreases (pre-administration vs. 9 h post): (i) Val-Tyr (228.2 ± 3.4 vs. 206.7 ± 9.5 mmHg) (*p* < 0.05) (ii) Ile-Tyr (205.6 ± 5.2 vs. 184.3 ± 4.5 mmHg) (*p* < 0.05) (iii) Phe-Tyr (208.7 ± 4.4 vs. 193.0 ± 5.1 (*p* < 0.01) (iv) Ile-Trp (213.3 ± 3.4 vs. 199.5 ± 5.9) (*p* < 0.05)	[229]
* *U. pinnatifida*	Enzymatic (Pepsin)	(i) Ala-Ile-Tyr-Lys (ii) Tyr-Lys-Tyr-Tyr (iii) Lys-Phe-Tyr-Gly (iv) Tyr-Asn-Lys-Leu	ACE-I inhibition IC_50_ values *in vitro*:((i) 213 μM (ii) 64.2 μM (iii) 90.5 μM (iv) 21.0 μM	[230]
* *P. palmata*	Enzymatic (Protease)	Ser-Asp-Ile-Thr-Arg-Pro-Gly-Gly-Asn-Met	Antioxidant activity after simulated gastrointestinal digestion: Oxygen radical absorbance capacity 152.43 ± 2.73 nM Trolox equivalents (TE)/µmol peptide and ferric reducing antioxidant power activity 21.23 ± 0.90 nM TE/µmol peptide,	[231]

* = *in vitro* studies; ** = *in vivo* animal studies; ^†^ = *in silico* studies.

**Table 4:** Seaweed-derived peptides and significant effects observed in intestinal epithelial cells *in vitro*.

Peptide	Seaweed	Study Type	Statistically Significant Effects	Ref.
* Ala-Leu-Glu-Gly-Gly-Lys-Ser-Ser-Gly-Gly-Gly-Glu-Ala-Thr-Arg-Asp-Pro-Glu-Pro-Thr	*P. yezoensis*	*In vitro* rat intestinal epithelial cells—investigating the modulation of cell differentiation.	At concentrations of 125–1000 ng/mL, the peptide, dose-depenently (*p* < 0.05): ▪ Induced intestinal epithelial cell proliferation ▪ Upregulated insulin receptor substrates IGF-IR, IRS-1, Shc and PY-99 ▪ Increased mRNA expression of p110, PDK1, p-Akt, c-Jun, c-Fos, and MAPK protein ERK1/2	[244]
* Ala-Leu-Glu-Gly-Gly-Lys-Ser-Ser-Gly-Gly-Gly-Glu-Ala-Thr-Arg-Asp-Pro-Glu-Pro-Thr	*P. yezoensis*	*In vitro* rat intestinal epithelial cells—investigating the epidermal growth factor receptor signalling pathway and Ras/Raf-p42/p44 MAPK signalling pathway, mediating signal transduction from cell surface to nucleus.	At concentrations of 125–1000 ng/mL, the peptide dose-dependently(*p* < 0.05): – Increased mRNA expression of p-EGFR, Shc, Grb2, SOS, Ras, Raf, mitogen activated extracellular kinase, and p-extracellular signal-regulated kinase. – Increased mRNA expression of p-EGFR, Shc, Grb2, SOS Ras, Raf, mitogen activated extracellular kinase, and p-extracellular signal-regulated kinase. – Increased mRNA expression of proteins required for cell proliferation: cyclin D1, cyclin E, Cdk2, Cdk4, Cdk6, and pRb – Increased cell growth during Gap 1 phase (47.6, 50.6, 56.8, 62.8 and 64.4% following treatment with 0, 125, 250, 500, and 1000 ng/mL of peptide, respectively) – Decreased mRNA expression of p21 and p27 associated with mucosal damage and ulcerative colitis.	[247]

* = *in vitro* studies.

**Table 5:** Advantages and limitations of gastrointestinal (GI) digestion model systems.

In Vitro Bioaccessibility Methods	Advantages	Limitations
**Solubility and Dialysability**	Simple and inexpensive to conduct with enzymes and dialysis filters that chemically mimic oral, gastric and small intestinal digestion Inexpensive No human or animal subjects required	Does not represent peristaltic movements, secretions, or gastric emptying of the GI tract No gut microbial component
**Static GI models**	Simple to conduct in single bioreactor or flask with stirring and pH adjustments Inexpensive No human or animal subjects required	Broad variance in results due to reagent diversity, particularly digestive enzymes which differ in activity dependent on their source (human, porcine, rabbit, bacterial, or fungal) Continuous mechanical agitation is not representative of complex peristaltic movements, secretions, or gastric emptying of the GI tract No gut microbial component
**INFOGEST static *in vitro* model**	Addresses worldwide lack of cohesion in simulated digestive methods Standardised static method suitable for food based on physiologically relevant conditions which can be applied for various endpoints Pepsin determined to be the factor causing most variation—activity determination may be improved by pH stabilisation Inexpensive No human or animal subjects required	Continuous mechanical agitation is not representative of complex peristaltic movements, secretions, or gastric emptying of the GI tract No gut microbial component
**Dynamic GI models**	Holistic *in vitro* gastrointestinal model incorporating the large and small intestine More representative of human GI digestion as changing physicochemical conditions and peristaltic forces are simulated in functionally distinct zones Human faecal inoculum included to study the effect of colonic fermentation on the food sample and nutrient absorption Samples can be taken at any stage of the digestive process without pausing the experiment Bioaccessibility results of dynamic models have been shown to correlate with bioavailability of the same nutrient *in vivo* No human or animal subjects required	More costly and lower throughput than static models Lack of *in vivo* factors such as first pass effect, renal clearance, and metabolisation by intestinal epithelial cells.
**Cell models**	Representative of intestinal epithelial cells Parallels human *in vivo* absorption studies May be used to mimic the ability of food components to be actively or passively transported and assimilated across the intestinal epithelium Human cell lines can be used as well as animal cells Mucus-producing cell lines can be co-cultured to more closely resemble *in vivo* conditions	Time-consuming to culture cell lines Costly First pass effect, renal clearance, interaction of the food sample with other nutrients and anti-nutrients, and different absorptive capacities at each stage of the gastrointestinal tract are not represented
***Ex vivo* bioavailability methods**	Multi-cell systems are more representative of intestinal epithelial behaviour in terms of food absorption than single cell lines Animal organ or tissue models can measure the oral bioavailability of bioactive food components Mimics arterial blood haemoglobin delivery by maintaining oxygen and carbon dioxide levels Precise measurement of electrical and transport parameters of intact epithelium Any type of intestinal epithelium from duodenum to colon can be studied, as well as other epithelia, such as the placental barrier No human subjects required	Extensive preparation Lack of inclusion of gut microbial influence Low throughput (mounted tissue models, such as Ussing chambers) Intestinal segment models have greater throughput, but no distinction between apical and basolateral side of the epithelium as tissue segments are fully submerged Short viability–epithelial intestinal tissue must be excised from animal within ~5 min of sacrifice Viability of intestinal tissues once the experiment begins is only ~150 min and not suitable for many oral bioavailability studies that require more time Limited range of measurements that do not fully describe the complex physiological system of the intestinal mucosa
***In vitro* fermentation models**	Static batch or dynamic fermentation models can be used Batch models are simple to set up and inexpensive Evaluates the impact of gut microbial populations on food bioaccessibility and bioactivity without using invasive human or animal methods Dynamic multistage models overcome the issue of fermentation product build-up in static batch models. pH and nutrient availability within each chamber are controlled throughout fermentation Computerised dynamic models such as TIM-2, SHIME and SIMGI create an anaerobic environment representative of the upper and lower digestive tracts rather than the colon alone in terms of bacterial populations and SCFA production Long-term stability of the microbiome—can be assessed as it adapts SHIME has option to set parameters found in diverse groups—humans, animals, diseased, healthy, elderly, or infants, and compare alternate treatments in parallel Possible to create a luminal or a mucosal microbiome Easier to obtain ethical approval compared to *in vivo* studies	Dynamic multistage models are costly and complex to set-up In static sealed batch models, fermentation products such as SCFA and p-cresol can accumulate and there is a finite amount of substrate available for the bacteria Lack of realistic peristalsis; expensive set-up costs; and absence of a dialysis component and mucosal cells (in the original SHIME model) Lack of realistic peristalsis in SHIME model and absence of a dialysis component and mucosal cells (in the original model) Lack of intestinal epithelial and immune cells in some systems. No feed-back mechanisms Use of parameters such as pH, redox potential, and transit time based on healthy individuals may not be representative of many groups
***In vivo* bioavailability methods**	Considered the gold standard and most accurate method for measuring bioavailability – analysis of metabolites in blood plasma and/or urine after a single dose, or controlled long-term consumption Reflects complete effect of digestion, first pass metabolism, Phase I/II biotransformation, host microbiota and fermentation on an orally consumed nutrient Balance studies collecting urine and stools to measure oral bioavailability are accurate Tissue distribution studies provide bioavailability data on the extent of absorption Data from *in vivo* studies is more clinically relevant and any side-effects induced by the consumed sample can be observed	Balance studies are laborious and more suited to laboratory animal models than human subjects Tissue distribution studies almost exclusively conducted on animals due to invasive nature Difficult to obtain ethical approval due to potential harm to animal or human participants and sacrifice of animals Usually more expensive and time-consuming than other methods Not suitable for high-throughput screening of bioavailability More difficult to control all variables due to naturally occurring differences in living organisms *In vivo* trials involving small cohorts may not be reflective of the bioavailability of a nutrient in the wider population

**Table 6:** Bioaccessibility of seaweed polyphenols.

Seaweed	Polyphenol	Extraction Method	Study Type	Observed Effects	Ref.
*; *** A. *nodosum*	Phlorotannins	Ethanolic crude phlorotannin extract (CE) and high-molecular-weight (HMW) fraction (>10 kDa) by tangential flow ultrafiltration. Combined as CE (58%) and HMW (42%)	(i) *In vitro* gastrointestinal enzymatic digestion, colonic fermentation, and dialysis to simulate absorption into the bloodstream. (ii) Cross-sectional human clinical trial (12 male, 12 female, healthy 18–65 years-old) (one capsule 101.89 mg phlorotannins). Blood and urine collected (0 to 24 h).	Phlorotannin metabolites detected in 15 of 24 participants after 24 h (total phlorotannins ranged from 0.011–7.76 μg/mL in blood plasma and from 0.15–33.52 μg/mL in urine).	[206]
*** A. *nodosum*	Phlorotannins	Ethanol CE extract and HMW fraction (>10 kDa) by tangential flow ultrafiltration. Combined as CE (57%) and HMW (43%)	24 week crossover study (8 weeks, 100 mg phlorotannin/d, or placebo capsule) (39 men, 41 women, mean BMI 30.2, mean age 42.7 years-old), 8 weeks washout phase, then repeat 8 weeks intervention or placebo treatment. Plasma and urine collected before/after each phase (0, 8, 16 and 24 weeks).	Polyphenol metabolites (0.5–11.8 mg/day total polyphenols) detected in 36 of 78 participants.	[358]

* = *in vitro* studies; *** = human dietary intervention studies.

**Table 7:** Bioactivity of seaweed peptides.

Seaweed	Peptide	Extraction Method	Study Type	Statistically Significant Effects Post-Digestion	Ref.
** U. pinnatifida	(i) Tyr-His (ii) Lys-Tyr (iii) Phe-Tyr (iv) Ile-Tyr	Hot water	*In vivo* study in spontaneously hypertensive rats. (a) Single oral administration of each dipeptide (50 mg/kg BM) (b) Continuous administration for 7 days (10 mg/day/kg BM)	(a) All dipeptides decreased (*p* < 0.05) blood pressure after single oral dose: Tyr-His decreased 50 mm Hg after 3 h Lys-Tyr decreased 45 mm Hg after 6 h Phe-Tyr decreased 46 mm Hg after 3 h IleTyr decreased Hg 33 mm Hg after 3 h (b) After 7 days continuous oral administration blood pressure was lowered (all *p* < 0.05 compared to pre-adminstraton): Tyr-His decreased 34 mm Hg Lys-Tyr decreased 26 mm Hg Phe-Tyr decreased 34 mm Hg IleTyr decreased 25 mm Hg Hypotensive effect of all four dipeptides lasted 3–8 weeks after ceasing continuous administration.	[374]
*; ** U. pinnatifida	(i) Ile-Trp (ii) Val-Trp (iii) Ile-Tyr (iv) Ala-Trp (v) Leu-Trp (vi) Val-Tyr (vii) Phe-Tyr	Enzymatic (Protease from *Bacillus stearothermophilus*) and HPLC separation to butanol-soluble fractions	(a) *In vitro* ACE-I inhibitory activity digestion stability study with pepsin, trypsin and chymotrypsin. (b) *In vivo* study in spontaneously hypertensive rats. Single oral administration of each dipeptide (1 mg/kg BM).	(a) No loss in ACE-I inhibitory activity post *in vitro* digestion.IC_50_ values: (i) Ile-Trp 1.5 µM (ii) Val-Trp 3.3 µM (iii) Ile-Tyr 6.1 µM (iv) Ala-Trp 18.8 µM (v) Leu-Trp 23.6 µM (vi) Val-Tyr 35.2 µM (vii) Phe-Tyr 42.3 µM (b) *In vivo* antihypertensive effect in spontaneously hypertensive rats (single oral dose, all 1 mg/kg of BW). Blood pressure decreases (pre-administration vs. 9h post): (i) Val-Tyr (228.2 ± 3.4 vs. 206.7 ± 9.5 mmHg) (*p* < 0.05) (ii) Ile-Tyr (205.6 ± 5.2 vs. 184.3 ± 4.5 mmHg) (*p* < 0.05) (iii) Phe-Tyr (208.7 ± 4.4 vs. 193.0 ± 5.1 (*p* < 0.01) (iv) Ile-Trp (213.3 ± 3.4 vs. 199.5 ± 5.9) (*p* < 0.05) Captopril control (238.7 ± 6.9 vs. 224.9 ± 4.1 (*p* < 0.05)	[229]

* = *in vitro* studies; ** = *in vivo* animal studies.
